# Mislocalized cytoplasmic p27 activates PAK1‐mediated metastasis and is a prognostic factor in osteosarcoma

**DOI:** 10.1002/1878-0261.12624

**Published:** 2020-02-14

**Authors:** Xiang Chen, Justin M. M. Cates, Yu-Chen Du, Antrix Jain, Sung Yun Jung, Xiao-Nan Li, John M. Hicks, Tsz‐Kwong Man

**Affiliations:** ^1^ Department of Pediatrics Baylor College of Medicine Houston TX USA; ^2^ Texas Children's Cancer Center Houston TX USA; ^3^ Department of Pathology, Microbiology, and Immunology Vanderbilt University Medical Center Nashville TN USA; ^4^ Advanced Technology Core Baylor College of Medicine Houston TX USA; ^5^ Mass Spectrometry Proteomics Core Baylor College of Medicine Houston TX USA; ^6^ Dan L. Duncan Comprehensive Cancer Center Baylor College of Medicine Houston TX USA; ^7^ Department of Pathology Baylor College of Medicine Houston TX USA

**Keywords:** biomarkers, metastasis, osteosarcoma, p27, PAK1

## Abstract

The development of pulmonary metastasis is the leading cause of death in osteosarcoma (OS), which is the most common malignant bone tumor in children. We have previously reported that the tumor suppressor p27 (KIP1, CDKN1B) is frequently mislocalized to the cytoplasm of OS. However, its prognostic significance and metastatic mechanism are still elusive. Here, we show that cytoplasmic p27 significantly correlated with a higher metastatic status and poorer survival of OS patients (*n* = 136, *P* < 0.05), highlighting the clinical significance of p27 mislocalization in OS. Mechanistically, cytoplasmic p27 is co‐immunoprecipitated with p21‐activated kinase 1 (PAK1), which resulted in higher PAK1 phosphorylations, actin polymerization, and cell motility in p27‐mislocalized OS cells. Silencing PAK1 expression in different p27‐mislocalized OS cell lines decreased the migratory and adhesion abilities *in vitro*, as well as the development of pulmonary metastases *in vivo*. Similar PAK1‐dependent motility was also observed in other p27‐mislocalized cancer cell lines. In summary, our study suggests that cytoplasmic p27‐mediated PAK1 activation is crucial for OS metastasis. A biomarker‐guided targeted therapeutic approach for metastatic OS and other cancers harboring p27 mislocalization can be developed, where cytoplasmic p27 is used for risk stratification and PAK1 can be exploited as a potential therapeutic target.

## Graphical abstract

Metastasis is the main cause of death in osteosarcoma (OS). In this study, we show that cytoplasmic p27 correlated with the prognosis of OS patients. p27 interacted and activated PAK1, which affected the migration, actin polymerization, cell adhesion, and metastatic abilities of OS cells. Our discovery is an important step toward developing a novel biomarker‐guided therapeutic approach for metastatic OS.

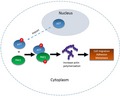

**Abbreviations**
DSS: disease‐specific survival
ECM: cell‐extracellular matrix
EFS: event‐free survival
IP: immunoprecipitation
mAb: monoclonal antibody
MEF: mouse embryonic fibroblast
MS: mass spectrometry
NES: nuclear export signal
OS: osteosarcoma
PAK1: p21‐activated kinase 1
RFP: red fluorescent protein
TMA: tissue microarray

## 1 Introduction

Osteosarcoma (OS) is the most common malignant bone tumor in children, adolescents, and young adults. Despite the remarkable advancements in adjuvant chemotherapy and limb‐salvage surgery, the survival of OS patients has not significantly improved over the last three decades. Metastasis remains a major cause of death in OS patients, but metastasis‐specific therapy is currently unavailable for the treatment of OS patients. Prior research efforts have focused on identifying drugs cytotoxic to OS cells, but the results have not been very rewarding (Khanna *et al.*, 2014). Recently, a growing consensus in the field is to intensify investigative efforts on discovering targets and drugs that can inhibit metastatic progression (Khanna *et al.*, 2014). The rationale is based on the new evidence that some metastatic tumor cells may remain clinically dormant in sanctuary organs and some can continue to spread to other parts of the lungs or other organs. Therefore, it is important to prevent metastatic progression in OS, even when the patients have already developed overt metastatic disease. Another challenge in the treatment of OS is the lack of molecular biomarkers to sensitively predict patients who are at a higher risk of developing pulmonary metastases. Conventional imaging techniques, such as computed tomography or magnetic resonance imaging, can miss small metastatic lesions. Because of the sensitivity of current imaging techniques, only 20% of OS patients have detectable metastases at diagnosis, while approximately 40% of the patients with localized disease at presentation eventually develop metastasis during or after therapy (Anderson, 2016). Hence, identification of a prognostic biomarker that not only identifies OS patients with a higher risk of distant metastasis but also represents a therapeutic target for inhibiting the development of metastasis would be of high clinical importance. A combination of a biomarker‐guided targeted therapy and conventional cytotoxic chemotherapy may offer better clinical outcomes for patients with metastatic OS.

To identify clinically useful prognostic biomarkers for OS, we have previously employed an immunoproteomic approach to analyze peripheral blood samples from a large cohort of OS patients. We demonstrated that a high level of autoantibodies against the tumor suppressor gene p27 (KIP1 or CDKN1B) is associated with poor outcomes in OS patients, while the p27 protein is frequently mislocalized to the cytoplasm of OS tumor cells (Li *et al.*, 2016). Ectopic expression of p27 in the cytoplasm increases the motility and invasiveness of OS cells *in vitro* and promotes the development of pulmonary metastases in mice (Li *et al.*, 2016). p27 is an atypical tumor suppressor that acts as a cyclin‐dependent inhibitor to control cell cycle progression of the G0‐S phase in normal cells (Slingerland and Pagano, 2000). However, it is rarely entirely deleted in human malignancies, suggesting that it may have a secondary function in cancer (Chu *et al.*, 2008). Recent studies have shown that cytoplasmic mislocalization of p27 occurs in many cancers; whereas loss of nuclear p27 or a gain of cytoplasmic p27 can be associated with poor prognoses (Denicourt *et al.*, 2007; Fukumoto *et al.*, 2004; Liang *et al.*, 2002; Rosen *et al.*, 2005; Singh *et al.*, 1998). However, the prognostic significance of p27 mislocalization in OS and other pediatric cancers is largely unknown.

Furthermore, the development of cytoplasmic p27‐specific targeting strategies has been challenging. Subcellular trafficking of p27 is regulated by major protein kinases, such as AKT and RSK1, via direct phosphorylation of the p27 protein. Because of the involvement of these kinases in multiple and diverse cellular signaling pathways, inhibition of these kinases would cause pleiotropic effects on tumor cells that may not be p27‐specific. At the downstream level, previous studies have also indicated that cytoplasmic p27 inhibits the RHOA‐GTPase activity, which can increase tumor cell motility (Besson *et al.*, 2004; Larrea *et al.*, 2009). In this study, we report the clinical utility of cytoplasmic p27 as a metastatic and prognostic biomarker for OS. We also discovered p21‐activated kinase 1 (PAK1) as a novel downstream effector of cytoplasmic p27‐mediated cell motility that may represent a therapeutic target in OS and other cancers that harbor p27 mislocalization. PAK inhibitors are under active development, and some of the inhibitors are being used in ongoing clinical trials (Semenova and Chernoff, 2017), suggesting that the results of this study may have a near‐term impact on the treatment of OS.

## 2 Materials and methods

### 2.1 Patient characteristics

Initial diagnostic (pretreatment) biopsy samples from 136 OS patients were represented on the tissue microarray used to study the association between cytoplasmic p27 and clinical outcome (metastasis and survival). The patient characteristics are tabulated in Table 1.

**Table 1:** Patient and demographic characteristics of the OS cases used in the tissue microarray analysis.

Characteristic	Time (year)	*n* (%)
All		136
Age (median; range)	17.6; 4.6–75.5	
Gender		
Female		69 (51)
Male		67 (49)
Race		
White		115 (85)
African American		17 (13)
Other		3 (2)
Asian		1 (< 1)
Histologic subtype		
Osteoblastic		52 (38)
Fibroblastic		32 (24)
Other		28 (20)
Chondroblastic		24 (18)
Site, *n* = 135		
Extremity		118 (87)
Other		18 (13)
Grade		
High		125 (92)
Low		8 (6)
Intermediate		3 (2)
Metastasis at diagnosis		
No		120 (88)
Yes		16 (12)
Histologic response, *n* = 115		
Good		73 (63)
Poor		42 (37)
Alive or dead		
Alive		73 (54)
Dead		63 (46)

### 2.2 Mice and human cell lines

Six‐ to eight‐week‐old NOD.CB17‐*Prkdc^scid^*/J mice (Jackson Laboratory, Bar Harbor, ME, USA) were housed in a pathogen‐free facility at Baylor College of Medicine. The NES‐p27 and phosphosite mutants used in this study were previously reported (Ahmed *et al.*, 2009; Li *et al.*, 2016). The Caki‐1 (HTB‐46) and MDA‐MB‐468 (HTB‐132) cell lines were purchased from American Type Culture Collection. HT‐1080 was a gift from N. Ahmed.

### 2.3 Mouse studies

2 × 10^6^ luciferase‐labeled 143B cells (Ahmed *et al.*, 2009) stably transfected with a PAK1 shRNA were injected into tail veins of 6–8‐week‐old mice. Ten mice were injected with the PAK1 shRNA mutant or with the scrambled shRNA control. The development of metastasis was monitored weekly by luminescence imaging (IVIS System; Xenogen, Alameda, CA, USA). Mice were sacrificed 4 weeks after injection to perform the histopathological examination of lung tissues to confirm the imaging results. An additional animal study was performed to investigate the effect of PAK1 silencing at early time points after tumor injection. The same amount of the scrambled control or the PAK1‐shRNA mutant was tail‐vein‐injected into a mouse. Luciferase imaging was used to monitor the location of injected tumor cells at different time points. After 24 h, the mice were sacrificed and lung tissues were resected and incubated with 150 µg·mL^−1^ of d‐luciferin for 5 min before imaging was performed.

### 2.4 Tissue microarray and immunohistochemical staining

Tissue microarray slides were deparaffinized with xylene, hydrated with water, and incubated at 95 °C for 20 min in antigen unmasking solution (Vector Lab, Burlingame, CA, USA). Then, the slides were incubated in 3% hydrogen peroxide for 10 min and normal horse serum (Vector Lab) for 30 min and subsequently incubated in mouse anti‐human p27 antibody (1 : 200; Santa Cruz, Dallas, TX, USA) at 4 °C overnight. Biotinylated horse anti‐mouse IgG antibody (1 : 200; Vector Lab) and the R.T.U VECTASTAIN Elite ABC Reagent (Vector Lab) were added sequentially for 1 h and 30 min, respectively. After color development with 3,3′‐diaminobenzidine solution (Vector Lab), the slides were dehydrated and mounted. p27 staining was evaluated and scored independently by a pathologist (JH). p27 immunostaining results were based on either the proportion score of positive cells with cytoplasmic p27 staining or the staining intensity score of cytoplasmic p27. The proportion score of positive cells was assigned as 0 when < 1% of stained cells were observed within the tumor, 1 when 1–25% of the tumor cells were positive, 2 when 26–50% of the tumor cells were positive, 3 when 51–75% of the tumor cells were positive, and 4 when > 75% of the tumor cells were positive. The intensity score was assigned as 0, 1, 2, and 3 for trace staining (background or minimal), weak staining, moderate staining, and strong staining, respectively. The total score was the summation of the proportion and intensity scores.

### 2.5 Fluorescent staining

For cytoskeleton staining, Alexa Fluor 594 Phalloidin (1 : 20; Thermo Fisher Scientific, Waltham, MA, USA) in 1% BSA/PBS was added for 1 h at 37 °C. For PAK1 immunofluorescent staining, anti‐human PAK1 antibody in 1% BSA/PBS was added for 1 h at 37 ^o^C. Anti‐rabbit IgG (H+L) F(ab′)_2_ fragment with Alexa Fluor 555 conjugate (1 : 50; Cell Signaling, Danvers, MA, USA) was added to detect PAK1. The slides were then mounted for Phalloidin staining and immunofluorescence detection by the Eclipse TE300 Microscope (Nikon, Minato, Tokyo, Japan), and images were collected using rs image software v1.9.2 (Roper Scientific, Tucson, AZ, USA).

### 2.6 shRNA‐mediated silencing of PAK1 expression

Two specific MISSION TRC Lentivirus shRNAs for PAK1 and an shRNA scramble nontarget control were used to generate PAK1‐silenced and control mutants in OS cell lines (MISSION^®^ Lentiviral Transduction Particles, shRNA#1 clone ID: TRCN0000195500, shRNA#2 clone ID: TRCN0000195636, and MISSION^®^ pLKO.1‐puro Non‐Mammalian shRNA Control Transduction Particles; Sigma‐Aldrich, St. Louis, MO, USA). Since our result showed that PAK1‐shRNA#2 was less efficient than PAK1‐shRNA#1, PAK1‐shRNA#1 was used to generate the silenced mutants in other non‐OS cancer cell lines.

### 2.7 Antibodies, subcellular fractionation isolation, and western blotting

Primary antibodies used in this study were anti‐human p27 (1 : 200; Santa Cruz), anti‐human PAK1 (1 : 1000; Cell Signaling), anti‐human phospho‐PAK1^S144^ (1 : 1000; Cell Signaling), and anti‐human phospho‐PAK1^T423^ (1 : 1000; Cell Signaling). Secondary antibodies used in this study were anti‐rabbit IgG, HRP‐linked antibody (1 : 3000; Cell Signaling) and anti‐mouse IgG, HRP‐linked antibody (1 : 3000; Cell Signaling). For subcellular fractionation and western blotting, nuclear and cytoplasmic fractions were prepared using NE‐PER nuclear and cytoplasmic extraction reagents (Thermo Fisher Scientific) according to the manufacturer's protocol. Anti‐human GAPDH (1 : 200; Santa Cruz) or tubulin (1 : 500; Santa Cruz), and HDAC1 (1 : 1000; Cell Signaling) were used as cytoplasmic and nuclear protein loading controls, respectively.

### 2.8 Cell proliferation and colony formation assays

A total of 1 × 10^4^ tumor cells in 100 µL culture medium were plated in a microtiter plate with five replicate wells for each cell line. Ten microlitre of CCK8 reagents was added for 1 h before measuring absorbance at 450 nm with the Multiskan FC Microplate Photometer (Thermo Fisher Scientific). Measurements from five consecutive days were used to generate cell growth curves. A total of 2 × 10^3^ tumor cells were cultured in three 6‐well plates for 7–14 days until cell colonies were formed (> 50 cells under a microscope). Then, the cell culture medium was removed and the plates were washed twice with 1× PBS. The colonies were incubated in 500 µL of Cell Stain reagents (Cell Biolabs) for 15 min at room temperature. Then, the plates were rinsed with water and air‐dried, and the number of colonies in the plates was counted.

### 2.9 Cell adhesion and migration assays

The CytoSelect 24‐well Migration Assays (Cell Biolabs, San Diego, CA, USA) were used to measure the migratory capabilities of OS and other cancer cell lines. 3 × 10^5^ cells grown in a serum‐depleted medium for overnight were added to the top chamber of a transwell. Then, the culture medium containing 10% FBS was added to the bottom well. After 4–6 h of incubation, migrated cells were stained by the Cell Stain Solution, which contains crystal violet. The numbers of migrated/invaded cells in five random fields under a microscope were counted in each condition/cell line, and the results were analyzed by two‐sample, two‐sided Student's *t*‐test. For the cell adhesion assay, 96 wells were coated with or without 10% Matrigel (BD Biosciences, San Jose, CA, USA), 10 µg·mL^−1^ fibronectin, and 10 µg·mL^−1^ collagen type I (Millipore, Burlington, MA, USA). Then, 1 × 10^5^ cells per well were plated and incubated for 30 min, and then washed twice with PBS. The cells were incubated with the Cell Stain Solution (Cell Biolabs) for 15 min, washed, and dried. One hundred microtiters of the Elution Buffer (Cell Biolabs) was added, and the absorbance at 520 nm was measured using the Multiskan FC Microplate Photometer (Thermo Fisher Scientific).

### 2.10 PAK1 inhibition by small‐molecule inhibitors

The PAK1 small‐molecule inhibitor FRAX‐597 (S7271) was purchased from Selleck Chemicals (Houston, TX, USA), and G‐5555 (HY‐19635) was purchased from MedChemExpress, LLC (Monmouth Junction, NJ, USA). DMSO was used as a vehicle control. For drug efficacy evaluation, 1 µm of FRAX‐597 or G‐5555 was incubated with the cell lines for 3 h. For migration assays, 1 µm of each drug was incubated with the starved cells before the assay. Migrated cells were stained and counted after 3 h of incubation.

### 2.11 CellLight actin‐RFP analysis

OS cells were incubated with 8–12 μL of CellLight Actin‐GFP, BacMam 2.0 reagent (Thermo Fisher Scientific) overnight for transduction. Transduced cells were then replated to new culture plates, and fluorescent images of the tumor cells at different time points were captured with an Eclipse TE300 Microscope (Nikon) and rs image software v1.9.2 (Roper).

### 2.12 RAC1/CDC42 activation assay

The experiments were performed using the RAC1/CDC42 Activity Assay Kit (Millipore). OS cell lysates were incubated with 5 µL (25 µg) of RAC/CDC42 Assay Reagent (PAK‐1 PBD, agarose). The beads were then resuspended with 50 µL of 2× Laemmli buffer and subjected to SDS/PAGE and western blotting of RAC1 (anti‐human RAC1 antibody, 1 : 1000) and CDC42 (anti‐human CDC42 antibody, 1 : 250). Total lysates were used as total protein input controls.

### 2.13 Immunoprecipitation followed by mass spectrometry

NES‐p27 or empty vector control cells were grown in culture medium, and the resulting cell pellets were washed in PBS and then lysed in 10 volumes of hypotonic buffer (10 mm HEPES, 10 mm KCl, 0.1 mm EDTA, 0.1 mm EGTA). The cells were homogenized by douncer and monitored by DAPI stain under a microscope to verify completeness of cell lysis (> 95% cells broken). The lysates were centrifuged at 3000 ***g*** for 10 min to separate the insoluble fraction from the soluble cytosolic fraction. The cytosolic fraction was ultracentrifuged at 200 000 ***g*** for 20 min at 4 °C and incubated with 5 µg of the anti‐human p27 antibody (DCS‐72; Santa Cruz) for 1 h at 4 °C, followed by ultracentrifugation and incubation with protein A sepharose slurry (GE Healthcare Life Sciences, Pittsburgh, PA, USA) for 1 h. The beads were briefly washed with NETN buffer (50 mm Tris pH 7.3, 170 mm NaCl, 1 mm EDTA, 0.5% NP‐40), boiled in 2× NuPAGE LDS Sample Buffer (Life Technologies, Carlsbad, CA, USA), and resolved on 10% NuPAGE Bis‐Tris Gel (Life Technologies). Resolved proteins on the gel were visualized with Coomassie Brilliant Blue stain and excised into gel pieces according to their molecular weights. The individual gel piece was destained and subjected to in‐gel trypsin digestion (GenDEPOT, Katy, TX, USA). The tryptic peptides were resuspended in 10 mL of loading solution (5% methanol containing 0.1% formic acid) and subjected to nanoflow LC‐MS/MS analysis with a nano‐LC 1000 system (Thermo Scientific) coupled to an Orbitrap Elite Mass Spectrometer (Thermo Scientific). The peptides were loaded onto a ReproSil‐Pur Basic C18 (1.9 µm, Dr. Maisch GmbH, Ammerbuch, Germany) precolumn of 2 cm × 100 µm size. The precolumn was switched in line with an in‐house 5 cm × 150 µm analytical column packed with ReproSil‐Pur Basic C18 equilibrated in 0.1% formic acid. The peptides were eluted using a 75‐min discontinuous gradient of 4–26% acetonitrile/0.1% formic acid at a flow rate of 800 nL·min^−1^. The eluted peptides were directly electro‐sprayed into the mass spectrometer. The instrument was operated in the data‐dependent mode acquiring fragmentation under the direct control of xcalibur software (Thermo Scientific). Precursor MS spectrum was scanned at 375–1300 *m/z* with 120 000 resolution at 400 *m/z*, 5 × 10^5^ AGC target (50 ms maximum injection time) by Orbitrap. Then, the top 35 strongest ions were fragmented by collision‐induced dissociation with 35 normalized collision energy and 1.6 *m/z* isolation width and detected by Iontrap with 30 s of dynamic exclusion time, 1 × 10^4^ AGC target, and 100 ms of maximum injection time. The obtained MS/MS spectra were searched against the Target‐Decoy Human RefSeq Database in Proteome Discoverer 1.4 interface (Thermo Scientific) with the Mascot 2.4 algorithm (Matrix Science). The precursor mass tolerance was confined within 20 p.p.m. with fragment mass tolerance of 0.5 daltons and a maximum of two missed cleavage allowed. Dynamic modification of oxidation, protein N‐terminal acetylation, and destreak were allowed. The peptides identified from the Mascot result file were controlled at 5% false discovery rate and subjected to manual verifications for correct assignment.

### 2.14 Immunoprecipitation followed by western blotting

Immunoprecipitation (IP) assays were performed using a Pierce Classic IP Kit (Thermo Fisher Scientific). Twenty‐three microlitre of 100 μg·mL^−1^ rabbit anti‐human p27 (D69C12) mAb (Cell Signaling) was added to the mixture and incubated at 4 °C overnight to form an immunocomplex. Normal Rabbit IgG (Cell Signaling) was used as a negative control. The mixture was added to 30 µL of protein A/G agarose resin and incubated at 4 °C for 1 h with gentle mixing. The resin was washed thrice with 200 µL of the IP lysis buffer and once with 100 µL of 1× conditioning buffer. The p27 immunocomplex was eluted with 50 µL of 2× Laemmli buffer (Bio‐Rad, Hercules, CA, USA) with 20 mm DTT. The eluent (20 µL) was loaded and analyzed in an SDS/PAGE gel for western blotting with the mouse anti‐human PAK1 mAb (1 : 100; Santa Cruz) or the mouse anti‐human p27 mAb (1 : 200; Santa Cruz) as a primary antibody.

### 2.15 Statistical analysis

The p27 proportion scores were analyzed with respect to the metastatic status at diagnosis and during the 3 or 5 years of clinical follow‐up as well as the histologic response to cytotoxic chemotherapy by two‐sided Fisher's exact tests. Univariate and multivariate survival analyses were performed in R package (coxph) using Cox proportional hazard models; *P*‐values were calculated using Wald tests. The histologic response was categorized as good (≥ 90% tumor necrosis) or poor (< 90% tumor necrosis). Kaplan–Meier survival plots and 5‐year survival rates were generated using the *survminer* package in R. Event‐free survival (EFS) was defined as disease progression (distant metastasis or local recurrence) or disease‐related death. If a patient suffered from more than one event, the earliest event was used in the analysis. Disease‐specific survival (DSS) was defined as cancer‐related death. All statistical analyses were performed in spss (IBM, Armonk, NY, USA) or R. The *P*‐value of each of the phenotypic assays and the number of mice developing metastases in the animal study were calculated by two‐sided Student's *t*‐tests and Fisher's exact test, respectively. *P* < 0.05 was considered significant in all the analyses.

### 2.16 Study approval

Study protocols for the use of de‐identified human tissue specimens in this study were approved by the IRBs of the respective institutions. The samples were collected after informed consents were signed. The study methodologies conformed to the standards set by the Declaration of Helsinki. The mouse study was performed under an animal protocol approved by Baylor College of Medicine's Institutional Animal Care and Use Committee.

## 3 Results

### 3.1 Cytoplasmic p27 is associated with OS metastasis and survival

**Figure 1:**
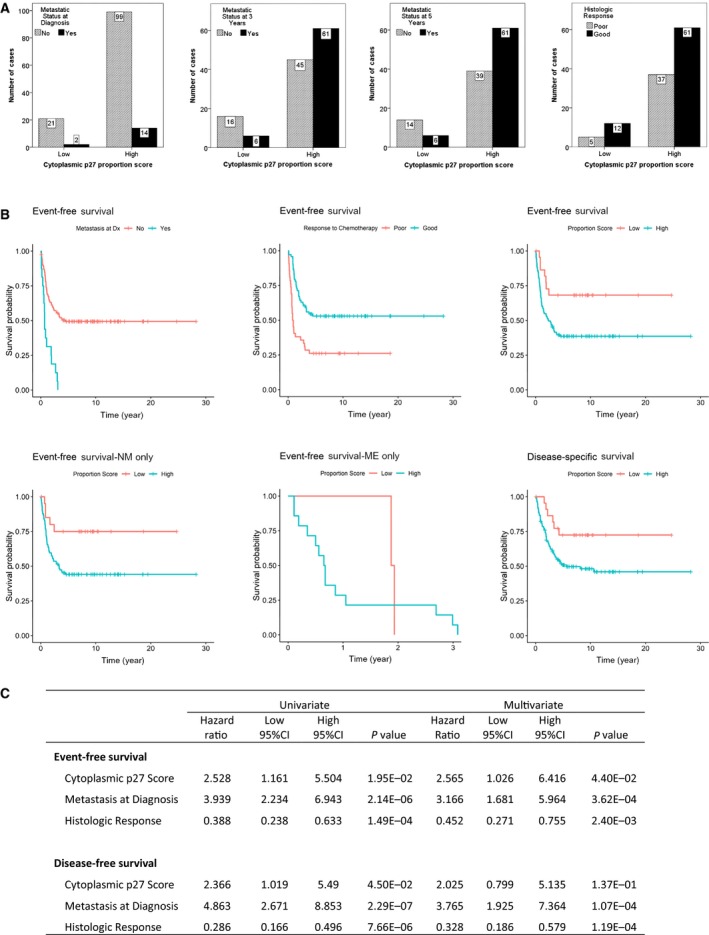
Correlation of clinical data with cytoplasmic p27 proportion scores in OS tissue microarray. (A) Bar graphs of the number of cases in different clinical categories by cytoplasmic p27 proportion score. Clinical categories from left to right are metastatic status at diagnosis, metastatic status at 3 years, metastatic status at 5 years, and histologic response to neoadjuvant chemotherapy. High cytoplasmic p27 score was significantly associated with development of metastatic disease within 3 years (*P* = 0.018) or 5 years (*P* = 0.014) of diagnosis. (B) Kaplan–Meier plots of EFS of OS patients. EFS was significantly associated with metastatic status at diagnosis (*P* = 2.14E‐06), histologic response (*P* = 1.49E‐04), and cytoplasmic p27 proportion score (*P* = 0.0195) (top left, middle and right). EFS of OS cases without metastasis at diagnosis (NM, *P* = 0.027, bottom left), but not in cases with metastasis at diagnosis (ME, *P* = 0.567, bottom middle), was significantly associated with cytoplasmic p27 score. DSS of OS cases was also significantly associated with cytoplasmic p27 proportion score (*P* = 0.045, bottom right). (C) Results of univariate and multivariate Cox proportional hazard model analyses for both EFS and DSS.

To examine the association between cytoplasmic p27 and OS metastasis, we conducted p27 immunohistochemistry on a clinically annotated tissue microarray (TMA) of OS (*n* = 136). All the specimens in the TMA were pretreatment biopsies collected at the time of diagnosis. Using cytoplasmic p27 proportion scores of the tumors, 23 (17%) OS cases were classified as ‘low’ and 113 (83%) cases were classified as ‘high’. These proportions were similar to those reported in our previous TMA study, suggesting that cytoplasmic mislocalization of p27 is a frequent post‐translational phenomenon in OS (Li *et al.*, 2016). Representative tumor images of the staining of different scores are shown in Fig. S1A. Additionally, representative TMA images for nuclear, cytoplasmic, and negative staining of p27 are shown in Fig. S1B. The cytoplasmic p27 proportion scoring was not significantly associated with demographic variables, such as patient age, gender, race, or histologic subtypes of OS (Table S1). However, the high cytoplasmic p27 scoring group was significantly associated with the presence of metastatic disease during 3‐year (*P* = 0.018) or 5‐year follow‐up (*P* = 0.014) (Fig. 1A), demonstrating for the first time a direct association between p27 mislocalization and the development of metastasis in OS patients. In contrast, no significant correlations were observed between cytoplasmic p27 proportion scoring and the metastatic status at diagnosis or the histologic response to neoadjuvant chemotherapy, the two major prognostic factors in OS (Fig. 1A). Since p27 mislocalization has been associated with poor survival in other malignancies (Chu *et al.*, 2008), we further evaluated the prognostic significance of p27 mislocalization in OS. The results of our survival analysis showed that metastasis at diagnosis and histologic response was significantly associated with EFS (Fig. 1B). The high cytoplasmic p27 scoring group also significantly predicted poorer EFS in OS patients (*P* = 0.0195, Fig. 1B). The 5‐year EFS rates of OS cases in the high and low cytoplasmic p27 scoring groups were 0.386 (95% CI, 0.306–0.488) and 0.682 (95% CI, 0.513–0.917), respectively. A multivariate Cox proportional hazard analysis revealed that the cytoplasmic p27 proportion scoring remained statistically significant after controlling for the metastatic status at diagnosis and the histologic response, indicating that it is an independent prognostic factor for EFS (*P* = 0.044, Fig. 1C). Similar results were observed when cytoplasmic p27 intensity scores (*P* = 0.037) or total scores (*P* = 0.015) were used in the EFS analysis (Fig. S2A). When only patients with localized disease at diagnosis were analyzed, the high cytoplasmic p27 scoring group still significantly predicted poorer EFS (*P* = 0.0270, Fig. 1B). However, the number of cases presented with metastases at diagnosis in our cohort was too small to derive a meaningful conclusion (only 3 out of 16 cases in the low p27 score group, Fig. 1B).

Besides EFS, the high cytoplasmic p27 scoring group was significantly associated with lower DSS (*P* = 0.045), with a 5‐year survival rate of 0.508 (95% CI, 0.422–0.612) in the high cytoplasmic p27 group and 0.724 (95% CI, 0.559–0.939) in the low cytoplasmic p27 group (Fig. 1B). While the two known prognostic factors also significantly correlated with DSS, cytoplasmic p27 was no longer significant for DSS after adjusting for metastasis at diagnosis and histologic response in a multivariate analysis (Fig. 1C and Fig. S2B).

### 3.2 Cytoplasmic p27 interacts with and activates PAK1

**Figure 2:**
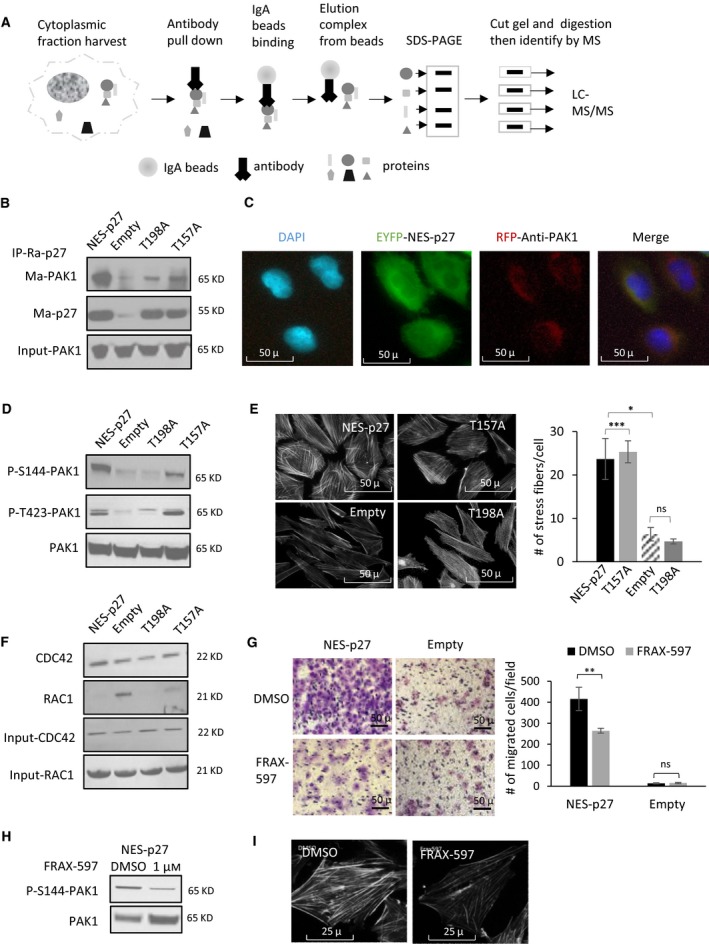
Characterization of the p27‐PAK1 interaction in OS cell lines. (A) Workflow of IP followed by mass spectrometry to identify cytoplasmic p27‐interacting proteins. (B) Results of p27 co‐IP with PAK1 in NES‐p27 cells, phosphosite mutants, and empty vector control. The pulldown assay was performed with rabbit p27 antibody (Ra‐p27), whereas PAK1 was detected by mouse PAK1 monoclonal antibody (Ma‐PAK1). Mouse monoclonal antibody p27 antibody (Ma‐p27) was used as an IP control, and total PAK1 expression was used as an input control. (C) Fluorescent images (20×) of cellular p27 and PAK1 showing p27 (EYFP, green) and PAK1 (immunofluorescence, red) proteins co‐localizing (merged, yellow) in the cytoplasm of NES‐p27 cells DAPI (blue) was used as nuclear counterstain. (D) Western blotting of phospho‐PAK1 in NES‐p27 cells, phosphosite mutants, and empty vector control. Total PAK1 protein was used as a loading control. (E) Images (left) and quantification (right) of phalloidin staining of F‐actin (stress fibers) in NES‐p27 cells, phosphosite mutants, and empty vector control (20×). For the quantification, experiments were repeated three times with ≥ 30 cells analyzed for each condition. (F) RAC1/CDC42 activity assays in NES‐p27 cells, phosphosite mutants, and empty vector control. RAC1 and CDC42 amounts of western blotting were used as input controls for RAC1/CDC42 activity assays. (G) Representative images (left) and quantification (right) of transwell migration assays of NES‐p27 and empty vector control cells treated with 1 µm of the Group I PAK inhibitor (FRAX‐597) or the vehicle control (DMSO). Migrated cells were stained, counted, and averaged using imagej software (National Institute of Mental Health, Bethesda, MD, USA) in five random and independent microscopic fields (10×). Error bars and asterisks represent standard deviations and statistical significance (Student's *t*‐test; *P < 0.05; **P < 0.01; ***P < 0.001, ns, not significantly, respectively). The experiment was replicated three times with a similar result. (H) Western blots showing amounts of phospho‐PAK1^S144^ and total PAK1 in the NES‐p27 mutant treated with FRAX‐597 or DMSO. (I) Representative images of phalloidin staining of actin stress fibers in NES‐p27 cells treated with FRAX‐597 or DMSO (40×).

To understand the metastatic mechanism of cytoplasmic p27, we seek to discover novel protein–protein interacting partners of cytoplasmic p27 in OS cells. We performed cytoplasmic p27‐specific IP followed by tandem mass spectrometry (MS) to compare the peptides/proteins pulled down in SaOS‐2 cells with (stably transfected with the NES‐p27 construct) or without (stably transfected with an empty vector control) cytoplasmic p27 (Fig. 2A). The NES‐p27 construct contains a nuclear export signal (NES) and an enhanced yellow fluorescent protein to ectopically express the p27 protein in the cytoplasm as previously described (Li *et al.*, 2016). The MS analysis discovered a total of 2,197 potential interacting proteins, while 247 of which were uniquely identified in NES‐p27 cells with at least two identifying peptides, but not in empty vector cells (Table S2). One of the top hits was PAK1, which is involved in tumorigenesis and metastasis (Radu *et al.*, 2014). The p27‐PAK1 co‐IP and MS results were subsequently confirmed by independent p27 IP followed by PAK1‐specific western blotting of NES‐p27 and empty vector cell lysates (Fig. 2B). We have previously shown that the NES‐p27 mutant harboring the T198A phosphomutation (NES‐p27^T198A^), but not the T157A phosphomutation (NES‐p27^T157A^), has lower cell motility than that of the NES‐p27 mutant (Li *et al.*, 2016). We, therefore, examined the relationship between the p27 phosphomutations and the p27‐PAK1 interaction using IP. The result showed that the amount of the PAK1 protein pulled down by p27 in NES‐p27^T198A^ cells was noticeably smaller than that in NES‐p27 or NES‐p27^T157A^ mutant cells (Fig. 2B), suggesting that the lower cell motility observed in NES‐p27^T198A^ mutant cells is due to a reduced p27‐PAK1 interaction. The result further indicates that the p27^T198^ residue in p27 is important for the protein–protein interaction. In addition to NES‐p27 cells, we also investigated whether the p27‐PAK1 interaction occurs in another sarcoma cell line (HT‐1080) with native cytoplasmic p27. The result showed that PAK1 was co‐immunoprecipitated with cytoplasmic p27 in HT‐1080 (Fig. S3A). The IP results were further supported by an *in situ* analysis of NES‐p27 cells, showing that the p27 (green) and PAK1 (red) proteins co‐localized (yellow) in the cytoplasm (Fig. 2C), ruling out that the interaction occurs inside the nucleus.

PAK1 is a serine/threonine‐specific protein kinase, which can autophosphorylate and activate itself during tumor development (Radu *et al.*, 2014). To determine whether the p27‐PAK1 interaction leads to PAK1 activation, we examined two major PAK1 phosphosites (S144 and T423) in NES‐p27 cells with and without p27 phosphomutations. Phospho‐immunoblotting results showed that the levels of phospho‐PAK1^S144^ in NES‐p27 and NES‐p27^T157A^ cells were higher than those in empty vector control and NES‐p27^T198A^ cells (Fig. 2D). Similar results were also observed for phospho‐PAK1^T423^, although the phosphorylation level was only moderately high in NES‐p27^T157A^ cells. The strong correlation between the PAK1 phosphorylation status and the p27‐PAK1 interaction in these OS cells suggests that the protein–protein interaction can lead to PAK1 activation, which can be regulated by the p27^T198^ phosphorylation. Since PAK1 can promote cell migration by influencing actin polymerization, we used phalloidin staining to visualize cellular F‐actin stress fibers. Actin stress fibers were significantly elevated in NES‐p27 and NES‐p27^T157A^ cells relative to empty vector and NES‐p27^T198A^ cells, consistent with their cellular phospho‐PAK1 levels (Fig. 2E). Nonetheless, PAK1 can be phosphorylated and activated by small RHO‐GTPases RAC1 and CDC42 (Edwards *et al.*, 1999). To investigate whether the cytoplasmic p27‐mediated PAK1 phosphorylations were manifested via indirect increase in the activities of these two GTPases, we performed RAC1 and CDC42 GTPase activity assays on NES‐p27 and its phosphomutant cells. The results indicated that the GTPase activities of CDC42 in various NES‐p27 mutants were similar to empty vector control cells, whereas the GTPase activities of RAC1 in NES‐p27 and the phosphomutant cells were lower than empty vector control cells, suggesting that the increased PAK1 phosphorylations in NES‐p27 cells are not an indirect upregulation of the GTPase activities of RAC1 and CDC42 (Fig. 2F).

To determine whether PAK1 activation was necessary for the increased motility observed in NES‐p27 cells and to evaluate whether the activation can be pharmacologically inhibited in OS cells, we conducted an inhibitor study using a commonly used Group I PAK inhibitor, FRAX‐597 (Licciulli *et al.*, 2013). The transwell assay results showed that the migration of FRAX‐597‐treated NES‐p27 cells, but not empty vector control cells, was significantly lowered when compared to that of DMSO‐treated cells, indicating that inhibition of PAK1 can reduce cytoplasmic p27‐mediated OS cell motility (Fig. 2G). We confirmed that FRAX‐597‐treated cells had a lower level of phospho‐PAK1^S144^ than DMSO‐treated cells (Fig. 2H). Consistent with the migration results, the amount of actin stress fibers was markedly decreased in NES‐p27 cells treated with FRAX‐597 (Fig. 2I). Besides FRAX‐597, similar migration inhibition results were also observed with another Group I PAK inhibitor, G‐5555 (Fig. S3B; Ndubaku *et al.*, 2015).

### 3.3 PAK1 silencing decreases tumor cell migration and adhesion, but not colony formation or proliferation

**Figure 3:**
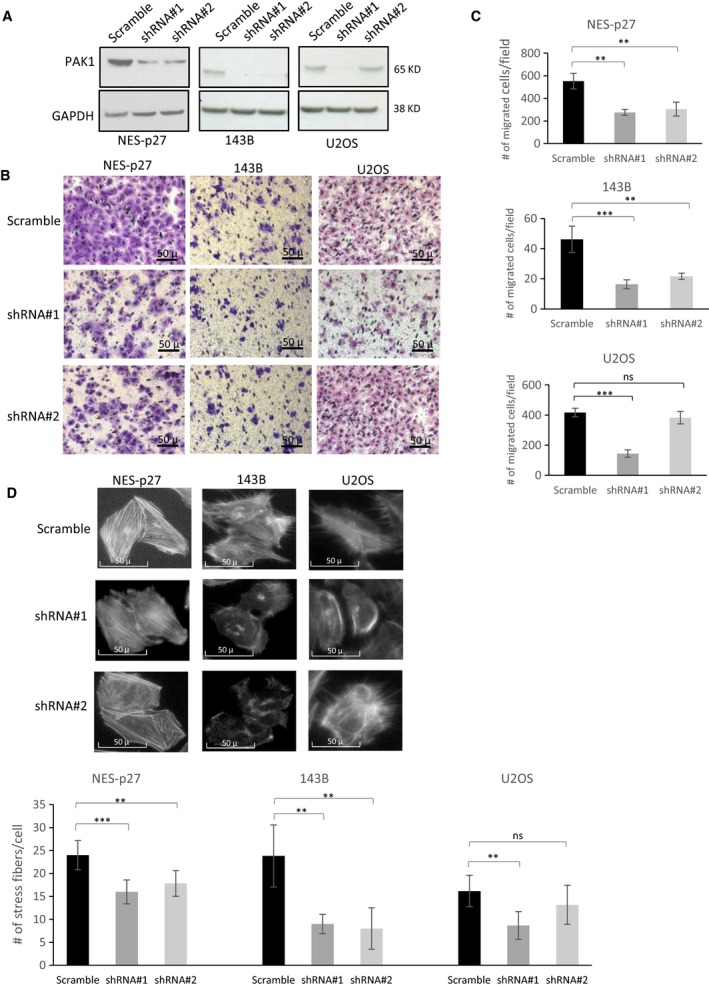
Effects of PAK1 silencing on tumor cell migration and actin stress fiber formation in three distinct OS cell lines with p27 mislocalization. (A) Western blots of total PAK1 in PAK1‐shRNA (shRNA#1 and #2) mutants and scramble controls from NES‐p27, 143B, and U2OS cell lines. (B) Representative images of the transwell migration assays of the two PAK1‐shRNA mutants and the scramble controls in the three OS cell lines. (C) Quantification of the transwell migration assays shown in B. Migrated cells were stained, counted, and averaged using imagej software in five random and independent microscopic fields (10×). The experiment was replicated three times. (D) Representative images and quantification of phalloidin staining showing the amount and distribution of actin stress fibers in the two PAK1‐shRNA mutants and the scramble controls in the three OS cell lines (20×). In the quantification analyses, error bars and asterisks represent standard deviations and statistical significance (Student's *t*‐test; *P < 0.05; **P < 0.01; ***P < 0.001, ns, not significantly, respectively).

Because of the potential nonspecific effects of Group I PAK1 inhibitors, we generated knockdown mutants in each of the three OS cell lines harboring p27 mislocalization (SaOS‐2/NES‐p27, 143B, and U2OS). Two PAK1‐specific shRNAs (shRNA#1 and shRNA#2) were used to rule out potential off‐target effects. NES‐p27 carries the ectopic expression of cytoplasmic p27, while the other two cell lines have a native p27 expression in the cytoplasm (Fig. S3C). The two PAK1‐shRNAs effectively knocked down PAK1 expression in the three OS cell lines, except the knockdown efficiency of shRNA#2 in U2OS was low (Fig. 3A). The result showed that the tumor cell migration of both shRNA mutants in NES‐p27 and 143B was significantly reduced when compared with the scramble controls, while the migration of U2OS was significantly reduced in the shRNA#1 mutant only, confirming the tumor cell migration was dependent on PAK1 expression and the migration results of the PAK inhibitor studies (Fig. 3B,C). Furthermore, the decrease in cell migration was consistent with lower amounts of actin stress fibers observed in PAK1‐silenced OS cells with low PAK1 expression (Fig. 3D). Our results also showed that PAK1 silencing did not alter cytoplasmic localization of p27 in the OS cell lines, suggesting that the antimigrational effect of PAK1 silencing is not mediated through altering p27 subcellular localization (Fig. S4).

**Figure 4:**
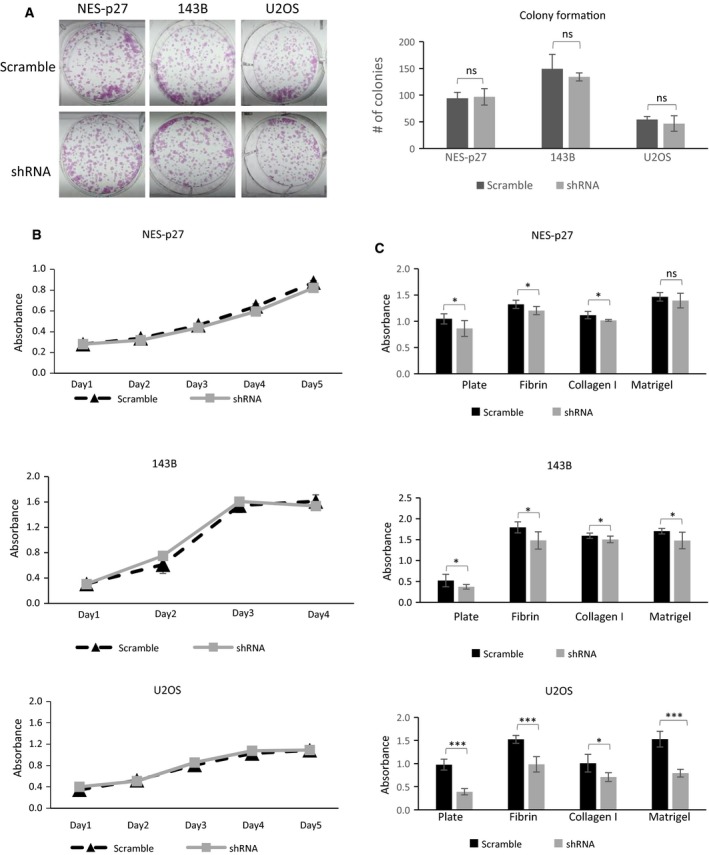
*In vitro* phenotypic analysis of PAK1‐shRNA#1 mutants derived from different OS cell lines. (A) Representative images and quantification of colony formation assays showing the tumorigenic potential of PAK1‐silenced mutants and scramble controls. (B) Growth curves of PAK1‐shRNA mutants and scramble controls. Five wells per cell were measured; error bars represent standard deviations of the replicates. Experiments were replicated three times. (C) Results of ECM adhesion assays to measure the adhesion abilities of PAK1‐shRNA mutants and scramble controls. The amounts of adhered cells were measured by *A*_560 nm_, and replicates were averaged per cell line for each of the indicated ECM components or the empty plate without precoated ECM component. Error bars represent standard deviations of the replicates, and asterisks denote statistical significance (Student's *t*‐test; *P < 0.05; **P < 0.01; ***P < 0.001, ns, not significantly, respectively). All experiments were replicated three times.

To further evaluate the effects of PAK1 on other metastasis‐associated processes, we measured colony formation, tumor cell proliferation, and tumor cell‐extracellular matrix (ECM) adhesion in the three PAK1‐shRNA#1 mutants and their scrambled shRNA controls. The phenotypic assays showed that there were no significant differences between the silenced mutants and their respective controls in colony formation (Fig. 4A) or cell proliferation (Fig. 4B). However, PAK1 silencing significantly impaired the adhesion ability of the three silenced mutants with or without different ECM substrates, including fibronectin, collagen I, and Matrigel (Fig. 4C). The only exception was when the Matrigel substrate was used; no significant differences were detected between the NES‐p27 and control cells. Together, these phenotypic analyses suggest that PAK1 is necessary for cytoplasmic p27‐mediated tumor cell migration and adhesion, but not colony formation or proliferation in OS cells.

### 3.4 PAK1 silencing reduces pulmonary metastasis *in vivo*

**Figure 5:**
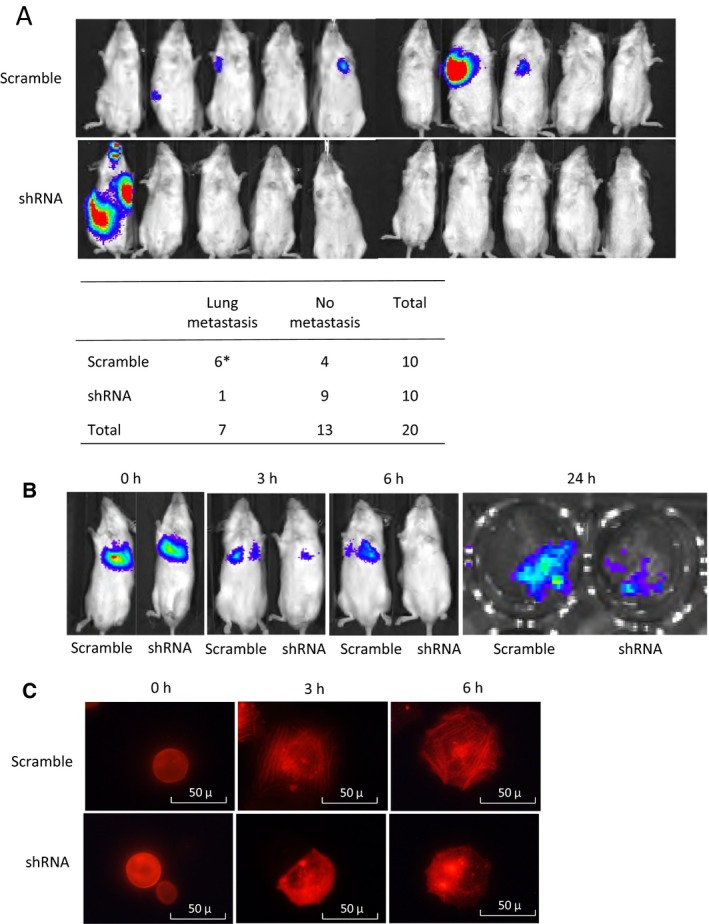
*In vivo* analysis of PAK1 silencing on OS metastasis. (A) Luminescence images of whole mice 4 weeks after tail‐vein injection of 143B PAK1‐shRNA#1 cells or scramble control cells. After an imaging analysis, mouse lungs were harvested and examined histologically to confirm the presence of pulmonary metastases. *One mouse in the scramble group developed micrometastasis in the lungs without detectable luminescence. Occurrence of metastasis was determined based on both luminescence and histopathological examination. The numbers of mice with or without metastases were compared between the shRNA and control groups (Fisher's exact test, *P* = 0.0286). (B) Luminescence images obtained early postinjection time points (0, 3, and 6 h) from mice injected with either the PAK1‐shRNA#1 or scramble control cells. The 24‐h image represents the luminescence image of the lung tissues harvested 24 h after tumor cell injection to illustrate tumor cell invasion of the lungs. (C) Representative fluorescence images of PAK1‐shRNA#1 and scramble cells at different time points (0, 3, and 6 h) after transduction with RFP‐actin in culture wells (20×). Experiments were replicated three times.

Based on the increased migration and ECM attachment phenotypes, we postulated that PAK1 activation may play a crucial role in tumor progression when OS cells acquire abilities to attach on to endothelial cells, extravasate from the circulation, and migrate within the pulmonary parenchyma. Thus, we adopted an experimental metastasis murine model that uses a tail‐vein injection method of tumor cells to specifically assess the effect of PAK1 silencing on the development of pulmonary metastasis *in vivo*. To detect the development of lung metastases, we employed luminescence imaging of tumor‐bearing mice followed by histologic confirmation of metastatic foci in the mouse lungs obtained at necropsy. The mouse study showed that the number of mice developing pulmonary metastases 4 weeks after the tumor cell injection was significantly lower in the PAK1‐shRNA group (1 out 10 mice) relative to the scrambled control group (6 out of 10 mice) (Fig. 5A). Similar results were also observed in HT‐1080 cells, where a PAK1‐silenced mutant produced significantly smaller pulmonary metastases relative to a scrambled shRNA control (Fig. S5).

Tumor cells adhere to the endothelial cells and extravasate into the lungs within hours after tail‐vein injection (Al‐Mehdi *et al.*, 2000; Tsuji *et al.*, 2006). To test whether PAK1 silencing affects these metastatic processes, we conducted a time‐course study to follow the injected tumor cells at early time points (0, 3, 6, and 24 h after tumor cell injection). The results indicated that both PAK1‐shRNA and scrambled control cells produced similar luminescence signals shortly after tail‐vein injection (0 h); however, the luminescent intensity of PAK1‐silenced cells decreased much more rapidly at 3‐ and 6‐h time points relative to that of scrambled control cells (Fig. 5B). The number of PAK1‐silenced cells extravasated into the lungs was much lower than that of scrambled control cells at 24 h (Fig. 5B). These results suggest that PAK1 may be important for the attachment of OS cells on to the endothelial cells of capillaries and extravasation. Using a red fluorescent protein (RFP)‐labeled transient actin expression system, we further demonstrated that the formation of actin filaments was significantly delayed in the PAK1‐shRNA mutant relative to the scrambled control within a similar time frame during the attachment of OS cells onto culture wells, suggesting the rapid clearance of OS cells in the mouse study may be associated with an impairment of actin filament formation (Fig. 5C).

### 3.5 PAK1 affects the motilities of other cancer cell lines with p27 mislocalization

**Figure 6:**
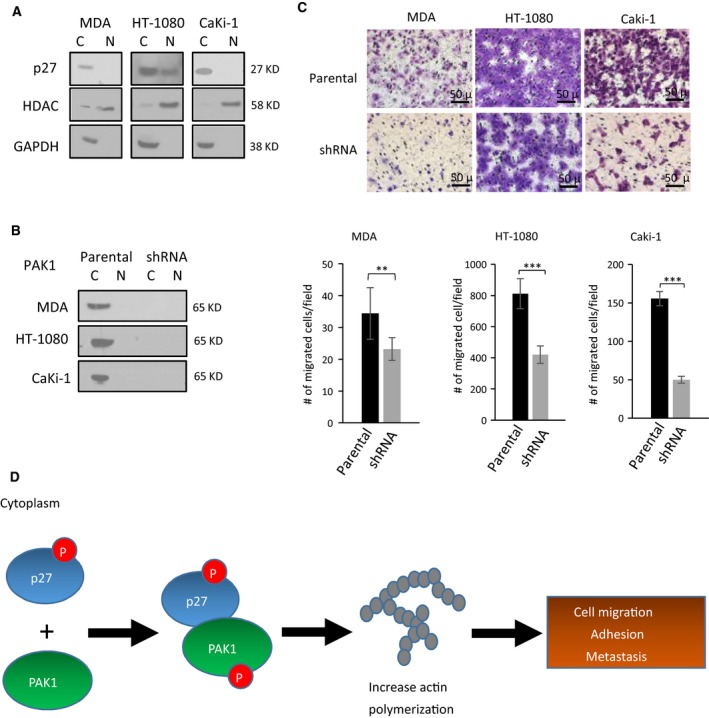
Migration inhibitory effect of PAK1 silencing on non‐OS cell lines with p27 mislocalization. (A) Western blotting with subcellular fractionation of three non‐OS cancer cell lines. GAPDH and HDAC were used as nuclear and cytoplasmic protein controls, respectively (C, cytoplasmic fraction; N, nuclear fractions). (B) Western blotting of PAK1‐shRNA mutants and parental controls in the three cancer cell lines showing high efficiency of PAK1 knockdown. (C) Representative images and quantification of transwell migration assays showing migrated cells in the three PAK1‐shRNA mutants relative to their parental cells. Migrated cells were stained, counted, and averaged using imagej software in five random and independent microscopic fields (10×). Error bars represent standard deviations of the replicates, and asterisks denote statistical significance (Student's *t*‐test; *P < 0.05; **P < 0.01; ***P < 0.001, ns, not significantly, respectively). All experiments were replicated three times. (D) A model depicting how p27 mislocalization may lead to increased incidence of metastatic progression in the OS patients. Phosphorylated p27 in the cytoplasm interacts with PAK1, and the resulting protein–protein interaction activates PAK1 by protein phosphorylation. The PAK1 phosphorylation promotes actin polymerization and increases stress fiber formation in OS cells, leading to higher tumor cell migration and adhesion and, hence, metastasis.

Previous studies have shown that p27 mislocalization occurs in multiple cancer types and the protein mislocalization correlates with poor prognoses (Ciaparrone *et al.*, 1998; Fukumoto *et al.*, 2004; Rosen *et al.*, 2005), but the role of PAK1 in these p27‐mislocalized cancers is still elusive. Using the breast cancer cell line MDA‐MB‐468, the fibrosarcoma cell line HT‐1080, and the renal cancer cell line Caki‐1, we showed that p27 was predominantly localized to the cytoplasm of the three cell lines (Fig. 6A; Kim *et al.*, 2009; Viglietto *et al.*, 2002). To examine the role of PAK1 in the three p27‐mislocalized cancer cell lines, we silenced the PAK1 expression in these cell lines (Fig. 6B). Using transwell migration assays, we demonstrated that all three PAK1‐silenced cell lines displayed significantly lower tumor cell migration than the parental cell lines (Fig. 6C). These results suggest that cytoplasmic p27‐mediated PAK1 activation may not be only important for OS cell migration, but also for the migration of other non‐OS cell lines with p27 mislocalization.

## 4 Discussion

Identification of a clinically applicable prognostic biomarker will likely improve the risk stratification of OS for therapeutic targeting. Our laboratory has previously reported that the p27 protein is frequently mislocalized to the cytoplasm of OS tumor cells. In this study, our results indicated that higher cytoplasmic p27 proportion scores were associated with metastases during the 3‐ or 5‐year follow‐up, but not at initial presentation. The insignificant result at initial presentation is likely due to the low number of OS patients with metastatic disease at diagnosis (Fig. 1A). Our survival analysis also indicated that OS patients with a higher proportion of OS tumor cells expressing the p27 protein in the cytoplasm have worse EFS (Fig. 1B). Similar results were observed when intensity scores or total scores were used, suggesting that both the protein expression level and the tumor cell proportion of cytoplasmic p27 are prognostically important. It is noteworthy that a low score cutoff was used in our study. Increasing the cutoff score did not improve the survival significance of cytoplasmic p27 (Fig. S2C). This finding suggests a highly detrimental effect of p27 mislocalization in OS because even cases with a low proportion of tumor cells with cytoplasmic p27 or low expression of cytoplasmic p27 can result in poor EFS. Furthermore, cytoplasmic p27 is an independent prognostic factor for EFS with respect to metastasis at diagnosis and histologic response, suggesting that it may augment the other two existing prognostic factors for more accurate prognostication of OS. Since cytoplasmic p27 did not retain its statistical significance in the multivariate analysis of DSS, the unique prognostic nature of cytoplasmic p27 may be primarily in predicting disease progression, and not necessarily biological aggressiveness (Fig. 1C). In addition, cytoplasmic p27 was not significantly associated with histologic response (Fig. 1A), suggesting that cytoplasmic p27 mainly influences the metastatic property, but not the intrinsic chemoresistance, of OS cells.

Currently, there are no well‐established molecular methods to predict which patients with a localized tumor at diagnosis are at a greater risk for distant metastasis or disease progression. Our survival analysis showed that cytoplasmic p27 can be used to identify a subgroup of localized patients who have a higher likelihood of developing clinical metastasis or relapses, suggesting that it can be used to augment conventional radiographic approaches by detecting the metastatic disease molecularly. In addition, cytoplasmic p27 can be easily detected by routine diagnostic immunohistochemical techniques used in most clinical pathology laboratories. Implementation of a p27‐based prognostic assay is highly feasible even in small or less equipped hospitals.

Benson *et al.* have previously found that re‐expression of p27 rescues the migrational defect of p27‐null fibroblast cells by reducing the RHOA‐GTPase activity. Lowering RHOA‐GTPase activity decreases the formation of stress fibers and focal adhesion complexes (Besson *et al.*, 2004). Consistently, we have previously shown that RHOA‐GTPase activity in NES‐p27 cells is downregulated in OS cells (Li *et al.*, 2016). Nonetheless, the current results showed that stress fiber formation was increased in highly mobile NES‐p27 and NES‐p27^T157A^ cells, which cannot be explained by the altered RHOA‐GTPase activity (Fig. 2). Hence, another factor may be involved in the regulation of actin stress fiber formation in NES‐p27 cells. We showed that PAK1 plays a critical role in p27‐mediated stress fiber formation in OS. First, PAK1 was activated in NES‐p27 and NES‐p27^T157A^ mutants, concomitant with an increased level of actin stress fibers in the cells (Fig. 2). Second, PAK1 silencing reduced actin stress fiber formation and *in vitro* metastatic phenotypes (tumor cell motility and cell adhesion), but not tumorigenic phenotypes (colony formation and cell proliferation) in three OS cell lines (Figs 3 and 4). Furthermore, PAK1 silencing produced significantly fewer mice that developed OS metastases (Fig. 5A). The effect of PAK1 may affect the ability of OS cells to establish in the metastatic niche, because PAK1‐silenced mutant cells were cleared much faster than control cells when OS cells were in the process of adhering onto capillary walls and extravasating into the lung tissues (Fig. 5B; Sahai, 2007). The timing of the clearance of OS cells at the metastatic site is consistent with the temporal delay of actin stress fiber formation in PAK1‐silenced cells, suggesting that PAK1‐mediated actin formation may play a role in the metastatic process (Fig. 5C). Together, our results suggest that PAK1 is critical for actin stress fiber formation and the migrational and adherence abilities of OS cells, which may then affect the adhesion between OS cells and endothelial cells and the extravasation process. Targeting PAK1 may be able to inhibit the formation of pulmonary metastasis even though OS cells have already entered the circulation.

PAK1 is a well‐known downstream effector of RAC1/CDC42, both of which belong to the family of RHO‐GTPases (Edwards *et al.*, 1999). Thus, p27 mislocalization may indirectly activate PAK1 by increasing the activities of the two RHO‐GTPases. Our results showed that PAK1 activation is not because of a higher activity of RAC1/CDC42. In fact, the RAC1 activity was decreased in NES‐p27 cells, suggesting a potential suppressive effect of cytoplasmic p27 on the RAC1 activity similar to that on RHOA (Besson *et al.*, 2004). The RAC1‐independent PAK1 activation is supported by a previous report that PAK1‐induced cytoskeletal changes are not mediated by RAC1 activation (Sells *et al.*, 1999). However, the precise regulatory mechanism of p27 mislocalization and various RHO‐GTPases will need to be further investigated. Since p27 is an intrinsically disordered protein (Galea *et al.*, 2008; Lacy *et al.*, 2004; Ou *et al.*, 2012) and PAK1 can autophosphorylate itself through conformational changes by interacting with other proteins (Chong *et al.*, 2001), one plausible model for the cytoplasmic p27‐mediated PAK1 phosphorylation is that cytoplasmic p27 binds to a yet to be identified PAK1 domain, which results in a PAK1 conformational change and autophosphorylation (Fig. 6D). Then, phospho‐PAK1 initiates actin polymerization to promote tumor cell motility and metastasis development.

A previous *in vitro* study has reported that PAK1 can act upstream of p27 to increase p27–cortactin interaction and PAK1‐mediated cortactin phosphorylation to promote the invasion of mouse embryonic fibroblasts (MEF) (Jeannot *et al.*, 2017). However, this particular mechanism is not supported by our cytoplasmic p27 IP–MS data, where cortactin was not co‐immunoprecipitated with cytoplasmic p27 in OS cells. The discrepancy may be due to the previous study mainly focused on the p27–cortactin effect on cell invasion in MEFs, while this study focuses on the p27‐PAK1 effect on cell motility and metastasis in OS. In addition, we have demonstrated the direct interaction of p27 and PAK1 and subsequent PAK1 activation in this study. The effect of PAK1 silencing on cytoplasmic p27‐mediated metastasis was also examined in a mouse model. Thus, we believe that our results are novel and provide the first and important mechanistic insight regarding how p27 mislocalization might promote metastasis in OS by interacting and activating PAK1‐mediated actin polymerization. This finding augments the results of our survival analysis by providing a plausible mechanistic explanation for why OS cases with cytoplasmic p27 tend to have poor outcomes and a higher risk of developing distant metastasis.

The results reported in this study may facilitate the development of a novel biomarker‐guided therapeutic approach for targeting OS metastasis. In this approach, OS tumors can be evaluated at diagnosis using conventional immunohistochemistry to determine the cytoplasmic p27‐mediated PAK1 activation status, and patients with the PAK1 activation would be treated with a PAK1 inhibitor. Our results showed that the Group I PAK inhibitor FRAX‐597 significantly decreased the migratory ability of NES‐p27 cells. The inhibitory effect on cell migration was confirmed by shRNA‐mediated PAK1‐specific gene silencing. Additionally, our mouse study showed that targeting PAK1 in OS cells reduced the development of pulmonary metastases. Although the murine model used does not recapitulate the entire metastatic process, it is arguably a more direct way of modeling the clinical characteristics of OS, where OS cells may have already left the primary site (Bruland *et al.*, 2005). Our findings suggest that despite the presence of OS in the lymphohematogenous circulation, PAK1‐specific therapy may still have a potential clinical benefit by reducing the progression of pulmonary metastases and the development of the clinically overt metastatic disease. The current study provides novel preclinical evidence and a rationale for the evaluation of PAK1 inhibitors in future OS clinical trials.

In addition to OS, p27 mislocalization has been found in other malignant neoplasms (Denicourt *et al.*, 2007; Kim *et al.*, 2009; Liang *et al.*, 2002). We showed that PAK1 inhibition reduced tumor cell migration in multiple types of cancer cells harboring p27 mislocalization, including fibrosarcoma, breast cancer, and renal carcinoma, suggesting that the cytoplasmic p27‐mediated PAK1 activation may be extended to other types of cancer. With the development of more specific and clinically applicable PAK1 inhibitors, future animal studies will be warranted to evaluate the therapeutic effects of PAK1 inhibitor strategies in various cancers. Notably, a recent clinical trial has been conducted using a PAK4 inhibitor, which also has a PAK1 inhibitory function (Rudolph *et al.*, 2015). Active pharmaceutical efforts are also in progress to identify more effective and specific small‐molecule inhibitors of PAK1 for cancer treatment (Radu *et al.*, 2014).

## 5 Conclusions

In summary, this study has high translational potential as we demonstrated that p27 mislocalization is a negative prognostic biomarker for OS and cytoplasmic p27‐mediated PAK1 activation is crucial for the development of metastasis in OS. Our findings also provide a basis for harnessing PAK1 as an actionable therapeutic target to inhibit the metastatic progression of OS and other cancers with p27 mislocalization.

## Conflict of interest

The authors declare no conflict of interest.

## Author contributions

XC performed the experiments with assistance from Y‐CD and X‐NL. AJ and SYJ executed the IP followed by mass spectrometry. JMMC developed the OS tissue microarray, and MJH scored the p27 immunohistochemistry. XC and T‐KM conceived the project, designed the experiments, and analyzed and interpreted the results. All the authors drafted and revised the manuscript.

## Supporting information


**Fig. S1.** Representative images of tumor cores on the tissue microarray.
**Fig. S2.** Kaplan‐Meier plots of cytoplasmic p27 in osteosarcoma tissue microarray.
**Fig. S3.** Additional experiments in p27 immunoprecipitation, migration assays and western blots of osteosarcoma cell lines.
**Fig. S4.** Subcellular fractionation followed by western blotting of p27 on PAK1 shRNA and scramble shRNA mutants from three osteosarcoma cell lines.
**Fig. S5.** Lung metastases of mice injected with HT‐1080 cells harboring PAK1 shRNA or scramble shRNA control.
**Table S1.** Comparisons of demographic factors and histologic subtypes with p27 proportion scores.
**Table S2.** Results of the p27 immunoprecipitation followed by mass spectrometry.
