# Supplementary material for: Mislocalized cytoplasmic p27 activates PAK1‐mediated metastasis and is a prognostic factor in osteosarcoma
Source: Mol Oncol. 2020 Feb 14;14(4):846–64. doi: 10.1002/1878-0261.12624 (PMC7138393; doi:10.1002/1878-0261.12624)
Supplement: Supplementary file 1 — Fig. S1. Representative images of tumor cores on the tissue microarray. Fig. S2. Kaplan‐Meier plots of cytoplasmic p27 in osteosarcoma tissue microarray. Fig. S3. Additional experiments in p27 immunoprecipitation, migration assays and western blots of osteosarcoma cell lines. Fig. S4. Subcellular fractionation followed by western blotting of p27 on PAK1 shRNA and scramble shRNA mutants from three osteosarcoma cell lines. Fig. S5. Lung metastases of mice injected with HT‐1080 cells harboring PAK1 shRNA or scramble shRNA control. Table S1. Comparisons of demographic factors and histologic subtypes with p27 proportion scores. Table S2. Results of the p27 immunoprecipitation followed by mass spectrometry. [file MOL2-14-846-s001.pdf]

Supplementary Figures and Tables

Chen *et al,* p27 mislocalization is a poor prognostic factor of osteosarcoma by activating PAK1-mediated metastasis

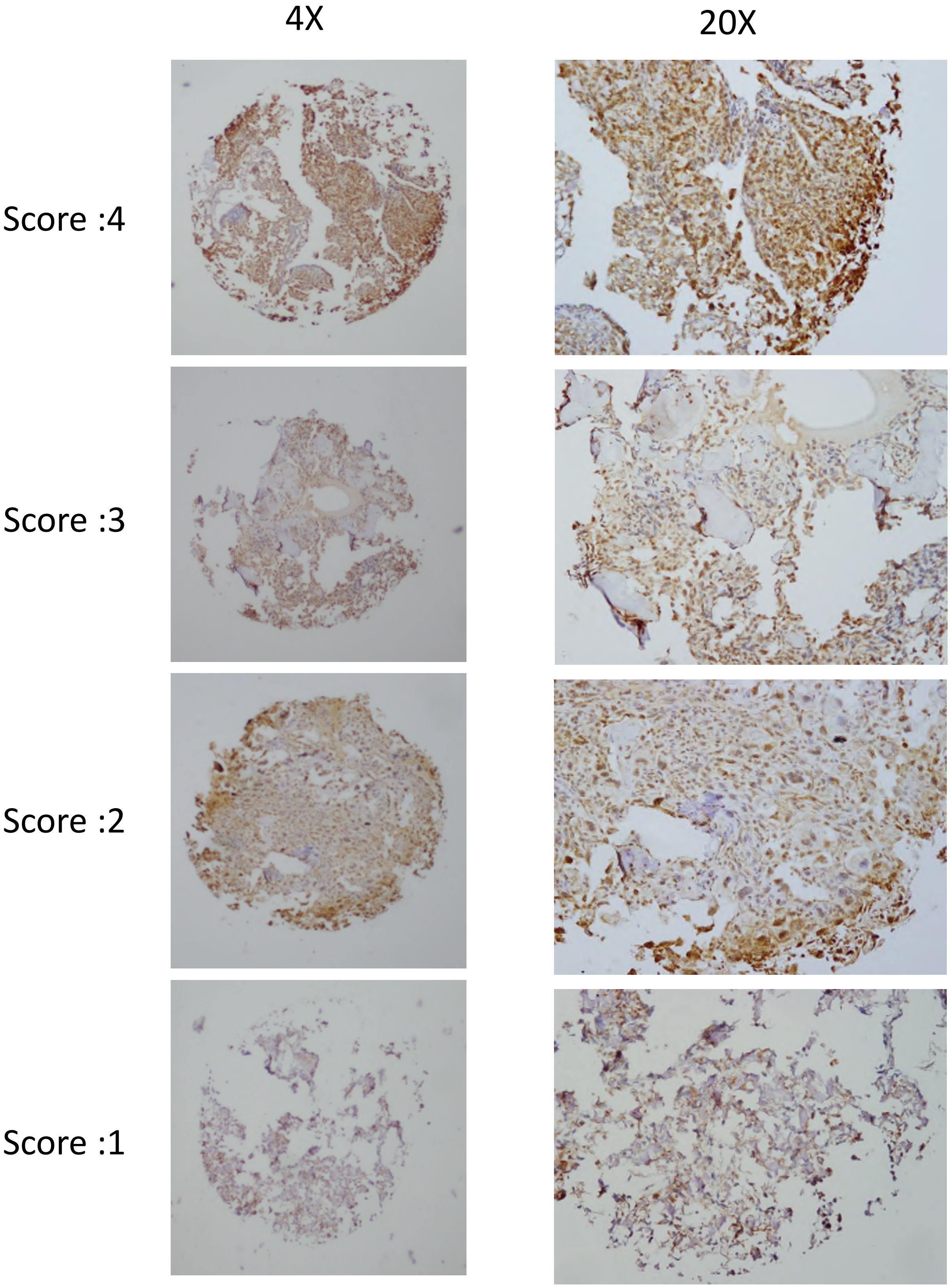

Supplementary Figure S1A. Representative images of tumor cores on the tissue microarray showing different proportion scores of p27 staining (brown) used in the survival analysis.

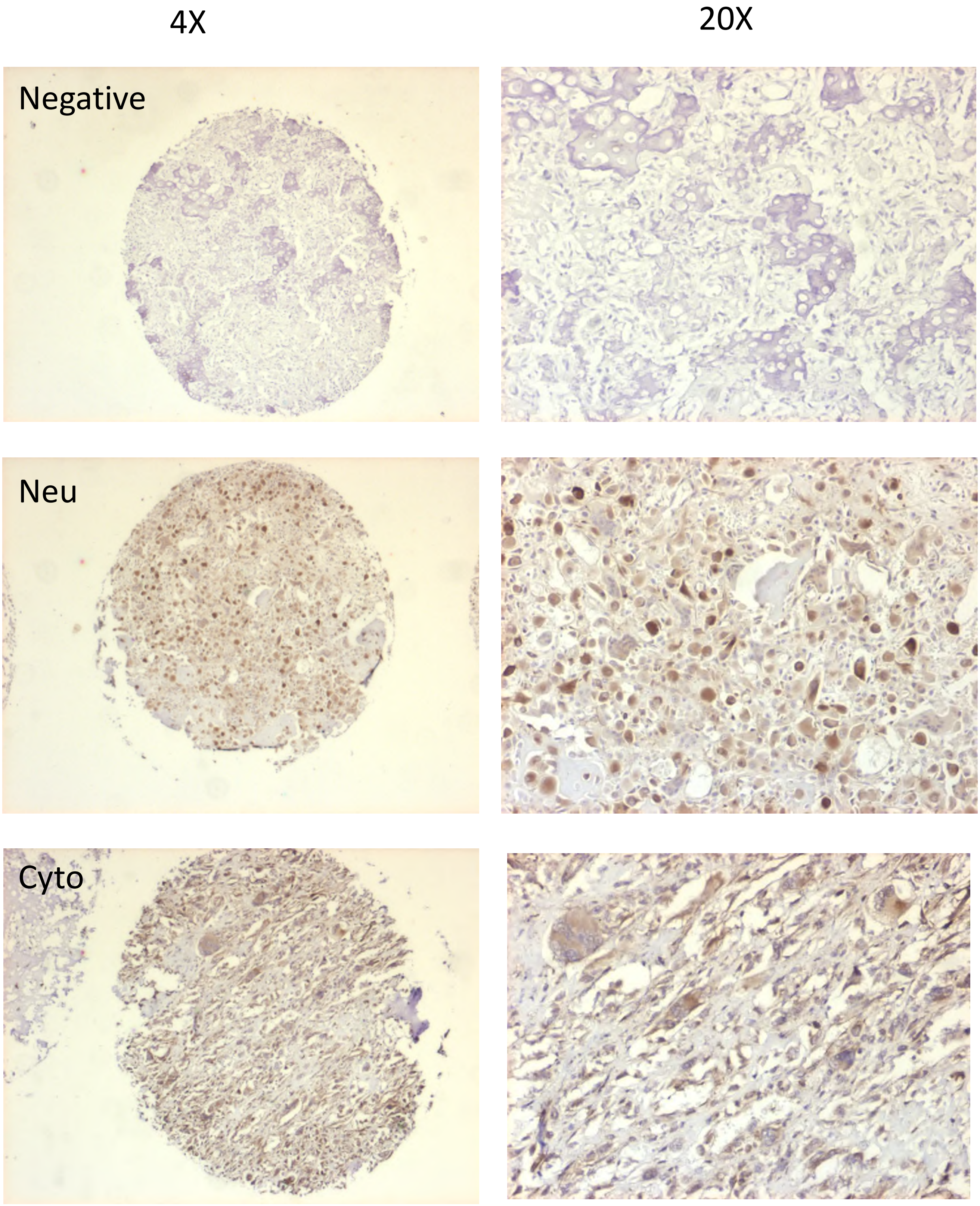

Supplementary Figure S1B. Representative images of tumor cores showing negative (Top), nuclear (Middle) and cytoplasmic (Bottom) IHC staining for p27 in the tissue microarray. Left and right columns show the lower (4X) and high (20X) power of magnification, respectively.

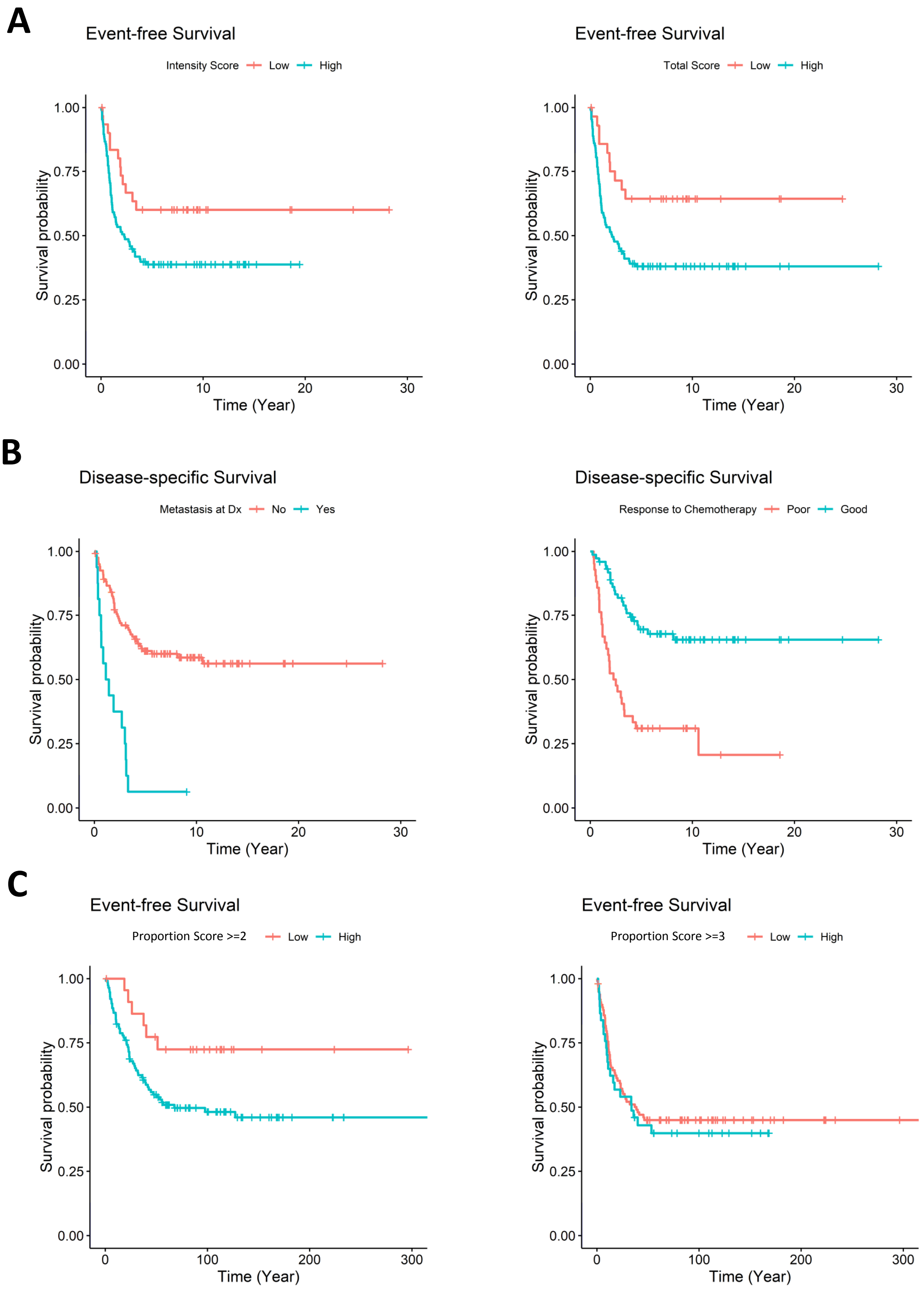

Supplementary Figure S2. Kaplan-Meier plots of cytoplasmic p27 in osteosarcoma tissue microarray. **A,** Event-free survival of intensity score (*p*=0.037, left) and total score (*p*=0.015, right) of cytoplasmic p27 in osteosarcoma cases. **B,** Disease-specific survival of metastasis at diagnosis (*p*=2.29E-07, left) and histologic response (*p*=7.66E-06, right) in osteosarcoma cases. **C,** Event-free survival of cytoplasmic p27 proportion score with cutoff >= 2 (*p*=0.10, left) and 3 (*p*=0.55, right).

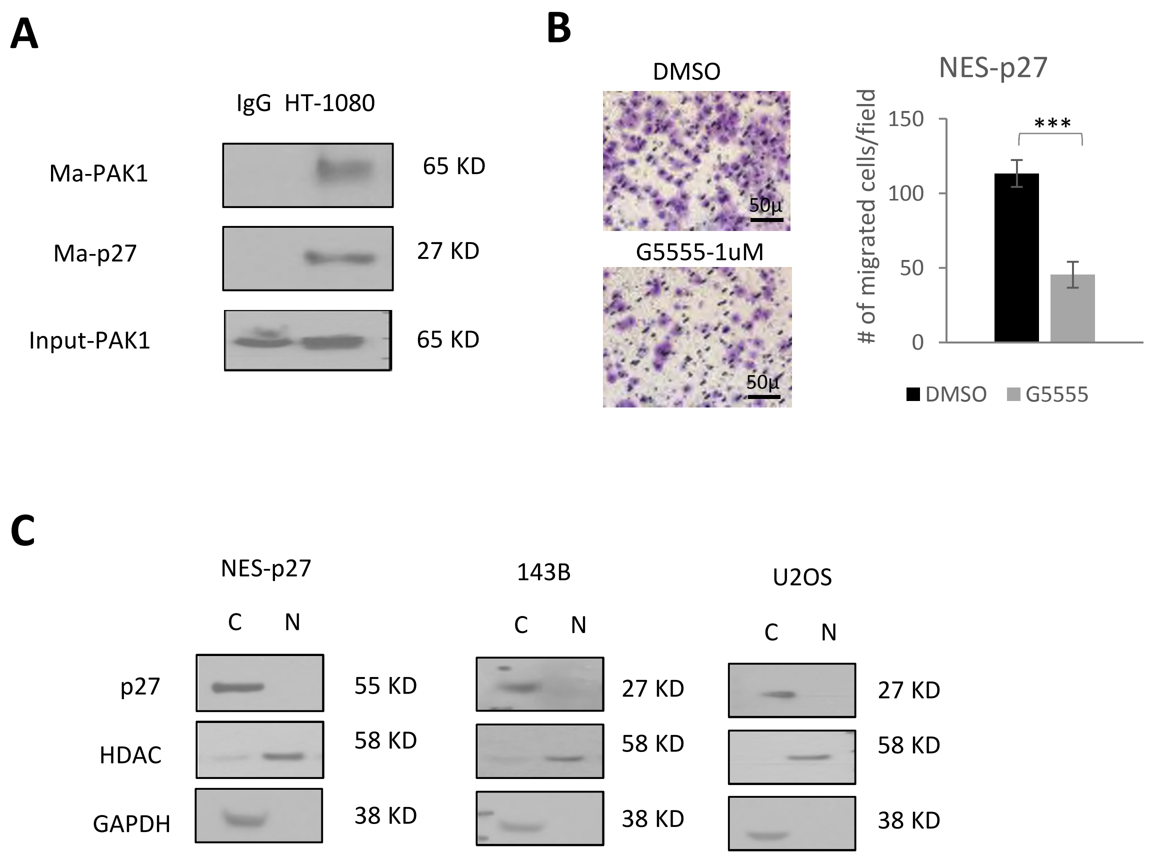

Supplementary Figure S3. A, Western blots of PAK1 in p27-immunoprecipitated proteins in HT-1080. The pulldown assay was performed by rabbit p27 antibody (Ra-p27), whereas same amount of cell lysis incubated with rabbit IgG was used as a negative control. PAK1 was detected by mouse PAK1 monoclonal antibody (Ma-PAK1). Mouse monoclonal antibody p27 antibody (Ma-p27) was used as an immunoprecipitation control and total PAK1 expression was used as an input control. **B,** Representative images (left) and quantification (right) of transwell migration assays of NES-p27 cells treated with 1µM of the Group I PAK inhibitor (G5555) or the vehicle control (DMSO). Migrated cells were stained, counted, and averaged using ImageJ 1.51K-IHC tool box (NIH) software in five random and independent microscopic fields (10X). Error bars represent standard deviations and asterisks denote statistical significance of the cell counts in the five fields (student-t test, $p<0.001$). The experiments were replicated three times. **C,** Fractionated Western blots of three OS cell lines (NES-p27, U2OS and 143B). HDAC and GAPDH were used as nuclear (N) and cytoplasmic (C) loading controls, respectively.

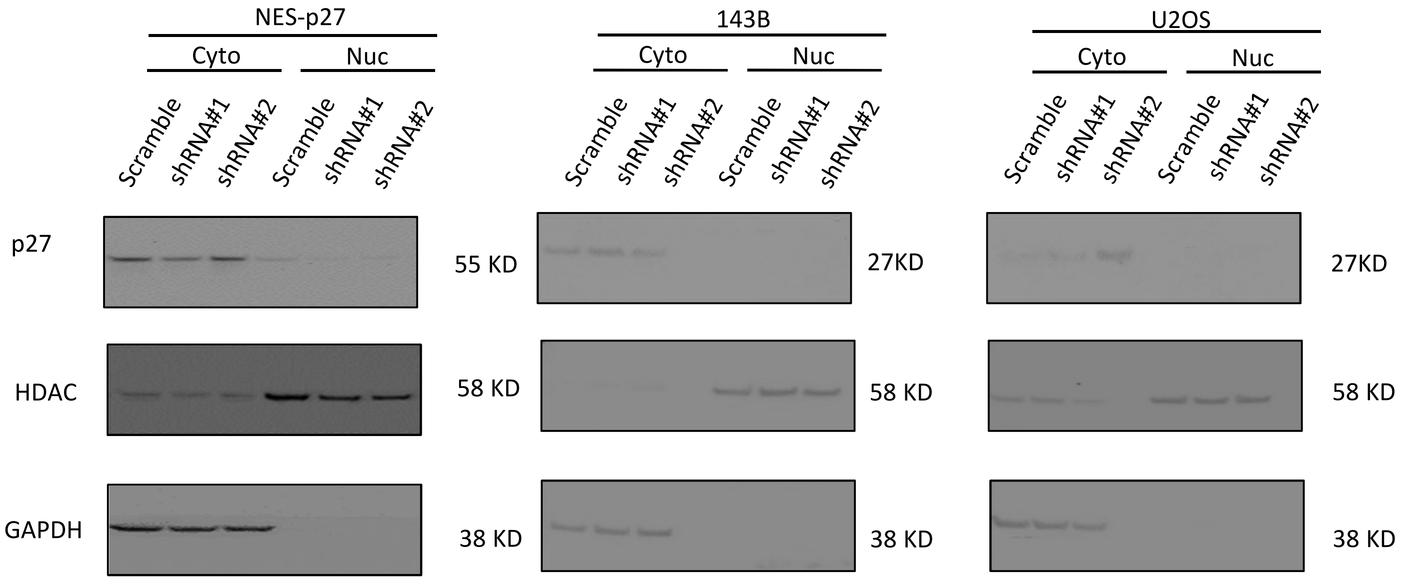

Supplementary Figure S4. Subcellular fractionation followed by Western blotting of p27 on PAK1 shRNA (shRNA#1 and #2) and scramble mutants of three osteosarcoma cell lines (NES-p27, 143B and U2OS). GAPDH and HDAC were used as cytoplasmic and nuclear protein controls, respectively.

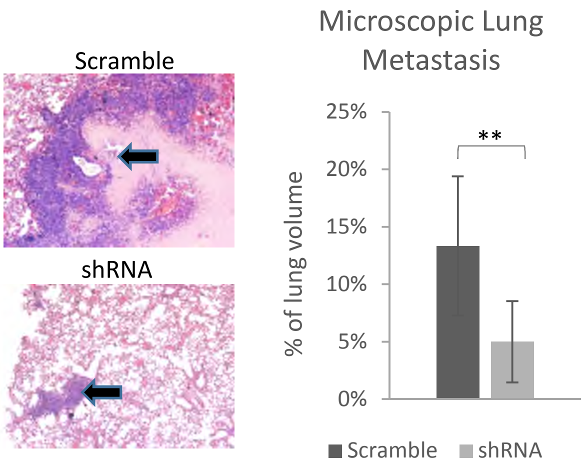

Supplementary Figure S5. Lung metastasis of mice injected with HT-1080 harboring PAK1-shRNA or scramble shRNA control. Four weeks after injection, mice were sacrificed and mouse lungs were harvested and examined by H & E staining to evaluate the presence of microscopic metastatic foci. Representative H & E staining images of metastatic nodules (20X) were shown (Arrow, left). The percentage of total lung volume occupied by tumor cells was counted and compared between the scramble and shRNA groups (Student's t-test, $p<0.01$, right).

Supplementary Table S1. Comparison of demographic factors and histologic subtypes with p27 proportion score. p-values for gender, race and histologic subtypes were calculated by 2-sided fisher-exact tests and p-value for age were calculated by a 2-sided student's t-test.

Demographics		p27 Proportion Score		*p*-value
		Low	High	
Gender				
	Female	12	57	1
	Male	11	56	
Race				
	African-American	0	17	0.068
	Caucasian	22	93	
	Other	1	3	
Histologic subtype				
	Chondroblastic	3	21	0.363
	Fibroblastic	5	27	
	Osteoblastic	7	45	
	Other	8	20	
Age (mean±stdev, year)		25±17	23±15	0.507

Supplementary Table S2. Results of the p27 immunoprecipitation followed by mass spectrometry to show cytoplasmic p27 interacting proteins uniquely present in the p27-mislocalized osteosarcoma cell line NES-p27 relative to the empty vector control. Only the proteins with at least two identified peptides are shown in the table. Cyclin-dependent kinase inhibitor 1B (p27) is shown in red and PAK1 is shown in bold.

Rank	NCBI gi no.	Protein Name	Peptide Hits	
			Empty Vector	NES-p27
	**4757962**	**cyclin-dependent kinase inhibitor 1B**	**26**	**62**
1	57013276	tubulin alpha-1B chain		150
2	171460916	dynamin-1-like protein isoform 2		48
3	119703753	keratin, type II cytoskeletal 6B		40
4	285395246	tubulin alpha chain-like 3 isoform 2		37
5	209969836	transportin-2 isoform 2		32
6	109148552	keratin, type II cytoskeletal 3		23
7	83641870	nucleophosmin isoform 3		22
8	38201625	eukaryotic translation initiation factor 4 gamma 1 isoform 3		21
9	419636284	AP-3 complex subunit beta-1 isoform 2		20
10	163310745	LIM and calponin homology domains-containing protein 1 isoform c		18
11	5453880	acidic leucine-rich nuclear phosphoprotein 32 family member A		17
12	6005743	ATP-dependent RNA helicase DDX19B isoform 1		16
13	21536301	signal transducer and activator of transcription 1-alpha/beta isoform beta (STAT1)		14
14	23111038	sorting nexin-2 isoform 1		14
15	112382257	inaD-like protein		13
16	24430146	nuclear pore complex protein Nup153 isoform 2		13
17	33469968	DNA replication licensing factor MCM7 isoform 1		12
18	28144897	adenylate kinase isoenzyme 5 isoform 1		12
19	13569879	acidic leucine-rich nuclear phosphoprotein 32 family member E isoform 1		12
20	459352730	POTE ankyrin domain family member J		11
21	53729363	E3 ubiquitin-protein ligase LRSAM1 isoform 1		11
22	372466577	keratin, type II cytoskeletal 8 isoform 2		11
23	33598968	LIM domain only protein 7 isoform 1		10
24	394582099	DNA replication licensing factor MCM3 isoform 2		10
**25**	**42794769**	**serine/threonine-protein kinase PAK 1**		**9**
26	6598323	rab GDP dissociation inhibitor beta isoform 1		9
27	39995059	caspase-2 isoform 1 preproprotein		9
28	15011972	rho guanine nucleotide exchange factor 1 isoform 2		9
29	289629267	protein transport protein Sec23B isoform 2		9
30	67191208	polyubiquitin-C		8
31	67782365	keratin, type II cytoskeletal 7		8
32	21536286	creatine kinase B-type		8
33	288915536	plastin-1		8
34	46249376	serine/threonine-protein phosphatase PP1-beta catalytic subunit isoform 1		8
35	5579478	dual specificity mitogen-activated protein kinase kinase 1		8
36	399154157	striatin-interacting protein 1 isoform 2		8
37	16753215	profilin-2 isoform a		7
38	21361091	ubiquitin carboxyl-terminal hydrolase isozyme L1		7
39	242247193	SH2B adapter protein 2		7
40	39930351	dynamin-binding protein		7
41	336176066	RNA-binding protein 39 isoform d		7
42	7524354	N(G),N(G)-dimethylarginine dimethylaminohydrolase 2		7
43	50083291	amyloid beta A4 precursor protein-binding family B member 2 isoform c		7
44	7019569	vacuolar protein sorting-associated protein 4A		7
45	351721296	protein SET isoform 3		7

46	215598688	ubiquitin carboxyl-terminal hydrolase 13	7
47	4504901	importin subunit alpha-4	7
48	21040371	ATP-dependent RNA helicase DDX39A	7
49	18375623	spliceosome RNA helicase DDX39B	7
50	50658065	structural maintenance of chromosomes protein 4	6
51	5803080	macrophage receptor MARCO	6
52	87578394	microtubule-associated protein 2 isoform 5	6
53	354681995	C-Jun-amino-terminal kinase-interacting protein 4 isoform 4	6
54	22538459	nuclear receptor coactivator 1 isoform 3	6
55	100913206	ATP-dependent RNA helicase A	6
56	166197690	ephexin-1 isoform 2	6
57	215490056	bifunctional protein NCOAT isoform b	6
58	347543829	dihydropyrimidinase-related protein 2 isoform 3	6
59	40316915	aminopeptidase B	6
60	282398114	breast cancer anti-estrogen resistance protein 1 isoform 2	6
61	198041728	kinesin light chain 2 isoform 2	5
62	94721263	myotubularin-related protein 12	5
63	40354195	keratin, type I cytoskeletal 18	5
64	222136585	protein timeless homolog	5
65	46909584	cAMP-dependent protein kinase catalytic subunit alpha isoform 2	5
66	190194412	thyroid receptor-interacting protein 11	5
67	7656952	calcyclin-binding protein isoform 1	5
68	194239668	SHC-transforming protein 1 isoform 4	5
69	4507783	ubiquitin-conjugating enzyme E2 H isoform 1	5
70	74027251	E3 ubiquitin-protein ligase TRIM33 isoform beta	5
71	10092601	eukaryotic translation initiation factor 4 gamma 3 isoform 3	5
72	156071452	nardilysin isoform b precursor	5
73	4759270	translin isoform 1	5
74	259906440	zinc finger CCCH-type antiviral protein 1-like	5
75	344030204	ras-related protein Rab-6A isoform c	5
76	5729980	phosphomevalonate kinase	5
77	20070376	putative sodium-coupled neutral amino acid transporter 10 isoform b	5
78	195546902	transcription elongation factor SPT5 isoform b	5
79	5031569	alpha-centractin	5
80	196114951	tyrosine-protein phosphatase non-receptor type 12 isoform 1	5
81	323276651	katanin p60 ATPase-containing subunit A1 isoform 2	5
82	23111041	sorting nexin-3 isoform b	5
83	62198241	protein CutA isoform 1	5
84	62198235	drebrin-like protein isoform b	4
85	6912604	acidic leucine-rich nuclear phosphoprotein 32 family member C	4
86	197209857	AP-3 complex subunit mu-2	4
87	23308579	prostaglandin E synthase 3	4
88	256600202	rap guanine nucleotide exchange factor 6 isoform 5	4
89	197333879	genetic suppressor element 1 isoform 2	4
90	289577109	ADP-ribosylation factor-binding protein GGA3 isoform 1	4
91	223671888	interleukin-1 receptor-associated kinase 4 isoform b	4
92	149274651	centrosomal protein of 78 kDa isoform b	4
93	56550081	mitotic checkpoint protein BUB3 isoform b	4
94	373838727	mini-chromosome maintenance complex-binding protein isoform 2	4
95	21493033	A-kinase anchor protein 10, mitochondrial precursor	4
96	66392203	NME1-NME2 protein	4
97	194733742	negative elongation factor A	4
98	106507301	DNA polymerase alpha catalytic subunit	4
99	381388773	amyloid beta A4 precursor protein-binding family B member 1 isoform c	4
100	359807035	TBC1 domain family member 1 isoform 2	4
101	6912478	importin subunit alpha-7	4
102	15529984	armadillo repeat-containing protein 6 isoform 2	4

103	19923592	oxysterol-binding protein-related protein 11	4
104	21541824	transducin-like enhancer protein 1	4
105	110578655	protein zwilch homolog	4
106	6912494	microtubule-associated protein RP/EB family member 1	4
107	109633028	serine/threonine-protein kinase MST4 isoform 2	4
108	7661914	26S proteasome non-ATPase regulatory subunit 6 isoform 2	4
109	20149496	EF-hand domain-containing protein D1 isoform 1	4
110	5802968	protein AF1q	4
111	4502011	adenylate kinase isoenzyme 1	4
112	21361837	PITH domain-containing protein 1	4
113	410171504	PREDICTED: hornerin	4
114	109255228	centrosomal protein of 170 kDa isoform alpha	4
115	397137355	PREDICTED: keratin, type II cuticular Hb3 isoform 2	4
116	58761500	obg-like ATPase 1 isoform 1	4
117	116008442	zinc finger CCCH domain-containing protein 13	3
118	209977117	netrin receptor UNC5D precursor	3
119	29789255	C-Maf-inducing protein isoform Tc-Mip	3
120	31657117	serine/threonine-protein phosphatase 4 regulatory subunit 3A	3
121	58530842	desmoplakin isoform II	3
122	237757326	synaptojanin-1 isoform d	3
123	194294525	intersectin-2 isoform 2	3
124	82659109	E3 ubiquitin-protein ligase UBR4	3
125	41327769	rho guanine nucleotide exchange factor 18 isoform a	3
126	384871702	DNA mismatch repair protein Msh2 isoform 2	3
127	112363080	microtubule-associated serine/threonine-protein kinase 2	3
128	21956645	myotrophin	3
129	148612885	ADP-ribosylation factor-like protein 2 isoform 1	3
130	281182700	WASH complex subunit FAM21C isoform 3	3
131	263191589	DNA mismatch repair protein Mlh1 isoform 2	3
132	49472841	A-kinase anchor protein 8-like	3
133	13491166	pumilio homolog 1 isoform 2	3
134	188528641	SEC14 domain and spectrin repeat-containing protein 1	3
135	22907034	folliculin isoform 1	3
136	332635087	phosphatidylinositol-binding clathrin assembly protein isoform 4	3
137	20070260	negative elongation factor B	3
138	71772415	40S ribosomal protein S15a	3
139	343887347	CD2 antigen cytoplasmic tail-binding protein 2	3
140	282165733	RUN and FYVE domain-containing protein 4	3
141	21361657	protein disulfide-isomerase A3 precursor	3
142	4506189	proteasome subunit alpha type-7	3
143	8922331	protein mago nashi homolog 2	3
144	216547519	interleukin-1 receptor-associated kinase 3 isoform a	3
145	303227928	matrin-3 isoform b	3
146	375151575	liprin-beta-2 isoform 3	3
147	304434687	serine/threonine-protein phosphatase 6 regulatory ankyrin repeat subunit B isoform B	3
148	27734925	uncharacterized protein C3orf33	3
149	296011003	abl interactor 1 isoform l	3
150	20070220	protein arginine N-methyltransferase 5 isoform a	3
151	4506711	40S ribosomal protein S27	3
152	386643034	protein NDRG1 isoform 3	3
153	4502749	cyclin-dependent kinase inhibitor 2A isoform p16INK4a	3
154	61742814	coiled-coil and C2 domain-containing protein 1B	2
155	11125770	aminoacyl tRNA synthase complex-interacting multifunctional protein 2	2
156	56549117	dynamin-1 isoform 2	2
157	4505913	mismatch repair endonuclease PMS2 isoform a	2
158	31742503	histone H3.2	2
159	120587025	SH3 and multiple ankyrin repeat domains protein 1	2

160	115430237	spectrin beta chain, non-erythrocytic 4 isoform sigma1	2
161	56550086	WD repeat and HMG-box DNA-binding protein 1 isoform 2	2
162	110347568	centrosomal protein of 152 kDa isoform 2	2
163	78217386	rab GTPase-activating protein 1-like isoform A	2
164	343403796	NEDD8 ultimate buster 1 isoform 2	2
165	145309300	cyclin-dependent kinase 13 isoform 2	2
166	110227613	arf-GAP with GTPase, ANK repeat and PH domain-containing protein 3 isoform a	2
167	217330559	serine/threonine-protein kinase ULK2	2
168	359338991	heterogeneous nuclear ribonucleoprotein Q isoform 7	2
169	5032087	splicing factor 3A subunit 1 isoform 1	2
170	41393563	kinesin-like protein KIF1B isoform b	2
171	112181295	xyloside xylosyltransferase 1	2
172	21389577	AP-4 complex accessory subunit tepsin	2
173	89363017	collagen alpha-2(V) chain preproprotein	2
174	209863002	MAP kinase-activating death domain protein isoform j	2
175	156766047	PERQ amino acid-rich with GYF domain-containing protein 2 isoform c	2
176	40548403	mRNA-decapping enzyme 1B	2
177	90819233	afadin isoform 2	2
178	57529246	ubiquitin carboxyl-terminal hydrolase 19 isoform 4	2
179	9966867	eukaryotic translation initiation factor 5A-2	2
180	221316716	proteasome subunit alpha type-1 isoform 3	2
181	156938331	pre-mRNA-processing factor 39	2
182	221219026	pleckstrin homology-like domain family B member 1 isoform b	2
183	33149331	nucleoredoxin isoform 1	2
184	296278204	partitioning defective 3 homolog isoform 10	2
185	47834348	minor histocompatibility protein HA-1 isoform 1	2
186	145301607	thyroid adenoma-associated protein isoform a	2
187	41327773	probable ATP-dependent RNA helicase DDX46	2
188	310110158	PREDICTED: otogelin isoform 1	2
189	387598060	guanine nucleotide-binding protein G(olf) subunit alpha isoform 2	2
190	7706457	A-kinase anchor protein 11	2
191	5802966	destrin isoform a	2
192	23943858	sorting nexin-8	2
193	22749199	fragile X mental retardation 1 neighbor protein	2
194	319890246	ropporin-1-like protein	2
195	5453557	E3 ubiquitin-protein ligase ARIH2	2
196	4502951	collagen alpha-1(III) chain preproprotein	2
197	41393573	ARF GTPase-activating protein GIT1 isoform 2	2
198	4758528	hepatocyte growth factor-regulated tyrosine kinase substrate	2
199	31881687	replication factor C subunit 4	2
200	226958663	nuclear pore membrane glycoprotein 210-like isoform 2 precursor	2
201	18379349	synaptic vesicle membrane protein VAT-1 homolog	2
202	4755140	type I inositol 3,4-bisphosphate 4-phosphatase isoform a	2
203	51988887	E3 ubiquitin-protein ligase SH3RF1	2
204	392494084	eukaryotic translation initiation factor 6 isoform a	2
205	166362732	ETS domain-containing protein Elk-1 isoform a	2
206	289577098	protein Shroom1 isoform 1	2
207	189458812	1,4-alpha-glucan-branching enzyme	2
208	87239981	tankyrase-1	2
209	23238222	COP9 signalosome complex subunit 3 isoform 1	2
210	73747879	WD repeat-containing protein 7 isoform 2	2
211	300796323	retinoblastoma-binding protein 5 isoform 2	2
212	82880674	cerebral dopamine neurotrophic factor precursor	2
213	188035877	probable ATP-dependent RNA helicase DDX59	2
214	110224465	GPI mannosyltransferase 3	2
215	315259084	integrator complex subunit 7 isoform 4	2

216	165905613	RNA-binding protein MEX3D isoform 1	2
217	4505585	platelet-activating factor acetylhydrolase IB subunit beta isoform a	2
218	24432026	kelch-like protein 22	2
219	4557367	bleomycin hydrolase	2
220	4557493	cleavage stimulation factor subunit 2	2
221	381214353	DIS3-like exonuclease 2 isoform 3	2
222	126722969	centromere protein T	2
223	166064025	cyclin-dependent kinase-like 3 isoform 2	2
224	7706497	UMP-CMP kinase isoform a	2
225	50428938	ATPase ASNA1	2
226	62460633	protein SCAF8	2
227	30089948	protein phosphatase 1E	2
228	15011936	40S ribosomal protein S26	2
229	21361559	visinin-like protein 1	2
230	23957690	conserved oligomeric Golgi complex subunit 7	2
231	37675283	AP-4 complex subunit epsilon-1 isoform 1	2
232	379056383	DNA polymerase delta subunit 2 isoform 1	2
233	61966721	tctex1 domain-containing protein 4	2
234	307574691	transforming growth factor beta receptor type 3 isoform b precursor	2
235	118402586	lactoylglutathione lyase	2
236	123173757	ribonucleoprotein PTB-binding 1	2
237	222418631	proton-coupled amino acid transporter 2	2
238	389565487	programmed cell death protein 6 isoform 4	2
239	13129078	tether containing UBX domain for GLUT4 isoform 1	2
240	217035118	TBC1 domain family member 23 isoform 2	2
241	306966160	leucine-rich repeat-containing protein 3C precursor	2
242	45359859	growth factor receptor-bound protein 2 isoform 2	2
243	4885413	histidine triad nucleotide-binding protein 1	2
244	10835049	transforming protein RhoA precursor (RhoA?)	2
245	151101404	prothymosin alpha isoform 2	2
246	485837026	spectrin beta chain, non-erythrocytic 5	2
247	39777614	calcium-responsive transactivator	2
