# Supplementary material for: Mislocalized cytoplasmic p27 activates PAK1‐mediated metastasis and is a prognostic factor in osteosarcoma
Source: Mol Oncol. 2020 Feb 14;14(4):846–64. doi: 10.1002/1878-0261.12624 (PMC7138393; doi:10.1002/1878-0261.12624)
Supplement: Supplementary file 2 [file MOL2-14-846-s002.pdf]

On the behalf of all the authors in this study, we thank you for the insightful comments and suggestions from the reviewer. We appreciate that the reviewer thought our manuscript is well written and the conclusions are strongly supported by our results. We have performed new experiments, added new figures, and revised the original figures and text to address each of the reviewer's comments as described below. We also rearranged the order of the images of the figures to make them consistent. We believe the revised version of this manuscript is much improved and hope that it is now acceptable for publication.

Comments to the Author

In the manuscript entitled "p27 mislocalization is a poor prognostic factor of osteosarcoma by activating PAK1-mediated metastasis", Chen and colleagues analyse the correlation between cytoplasmic p27 and survival of osteosarcoma patients. By using MS, authors identify PAK1 as a novel interactor of p27. Interestingly, this interaction apparently leads to the regulation of cytoskeleton rearrangements which are of pivotal importance in cell adhesion and metastasis development. The manuscript is well written and the conclusions are strongly supported by the data presented. To note, authors both use a broad range of cell lines and perform in vivo experiments (IHC/TMA and xenograft model). The manuscript is almost suitable for publication in Molecular Oncology however I have some issues to address:

1. Please, could you demonstrate the representative images of IHC from your TMA to make it easier to the reader to understand the scale of IHC intensity you are using (i.e. for each level of intensity show one representative image).

>> As the reviewer suggested, representative TMA core images of low (4X) and high (20X) magnification powers of different IHC staining scores (Proportion scores 1-4) used in the survival analysis have been included in Supplementary Figure S1A. Additionally, we have also included representative TMA images for nuclear, cytoplasmic, and negative staining of p27 (Supplementary Figure S1B).

2. Could you split the panel Fig 2B into three independent panels and put them in the order according to the order of results (i.e. 1: IP/IF, 2: WB, 3: phalloidin/GTPases).

>> The Fig. 2B panel is now divided into three separate panels according to the order of the description in the main text as suggested by the reviewer. The Fig. 2B and 2C contain the immunoprecipitation and immunofluorescence results, respectively. Fig. 2D contains the Western blotting results, Fig. 2E describes the

actin stress fibers, and the Fig. 2F contains the RHO-GTPase results. We believe that the revised Fig. 2 is much easier to read as the order the sub-figures follows the order of description in the main text.

3. Scale bars for images and MW for WB must be added, as well as uncropped versions for all WB must be provided (as a Supplementary file or only for revision).

>> The scale bars of all the cell images and the molecular weight markers of all the Western blotting results have been added in the revised Figures 2 and 3. The uncropped versions of the Western blotting results have also been included in this submission for review (Review Figure 1 attached).

4. On the Fig 3C the differences in the stress fiber number are not so evident thus I would not state that the migration experiments are strongly confirmed by phallodin staining. Probably, a quantification (as in the Fig 2) could help to make these differences more evident.

>> We agree with the reviewer that quantification of the Phalloidin staining will better discriminate the differences of the actin stress fiber results in Fig. 3C. Thus, we quantified the Phalloidin staining images using the ImageJ software and performed a statistical analysis (t-tests) of the quantification results. The new analysis showed that the stress fiber amounts in osteosarcoma cell lines was significantly lowered by shRNA-mediated PAK1 gene silencing ($p<0.05$). Since a new PAK1-shRNA mutant was created as described in Comment #6, new Phalloidin staining figures are added.

5. Could you perform the IF staining of p27 in these cell lines (Fig 3C)?

>> Since IF can only produce semi-quantitative results of the subcellular localization of p27 in osteosarcoma cell lines, we performed a more quantitative approach to analyze the p27 subcellular localization in the three osteosarcoma cell lines used in Fig. 3C to better address the reviewer's comment, i.e. subcellular fractionation followed by Western blotting. Nuclear and cytoplasmic protein controls were added. The same approach was also used to analyze the other cancer cell lines in Fig. 6A. The new results showed that p27 was predominantly localized in the cytoplasm of the three osteosarcoma cell lines. The new result is added in Supplementary Fig. S4.

6. Since it is broadly accepted that at least 2 si/shRNA must be used to avoid any possible off-target effect, it would be nice if you tried to use another shRNA for PAK1 in the migration assay / phalloidin staining.

>> We thank you for the reviewer’s suggestion to further improve our results. As suggested, we used another PAK1 shRNA (shRNA#2) to develop an additional PAK1-shRNA mutant in the three osteosarcoma cell lines. As indicated in the revised Fig. 3, the new shRNA efficiently reduced the PAK1 expression in NES-p27 and 143B cell lines, but not in U2OS, suggesting this new shRNA is not as commonly effective as the original shRNA (shRNA#1) used. We then examined the migration ability of the new PAK1-shRNA osteosarcoma mutants and found that the migration of the silenced mutant cells was significantly decreased in NES-p27 and 143B when compared with the scramble controls (Fig. 3B-C), ruling out that the decrease of tumor cell migration is due to off-target effects of the original PAK1-shRNA used. The migration of the new shRNA mutant in U2OS was similar to that in the scramble control, which is consistent with the low knock-down efficiency in this cell line. In addition, Phalloidin staining on the new shRNA mutants showed a consistent result that the actin stress fiber formation is affected by PAK1 expression. This new information has been added to the Result section of the revised main text. In addition, as indicated in our PAK1 inhibitor analysis, the tumor cell migration was also decreased in the FRAX-597 treated cells when compared with DMSO-treated cells (Fig. 2G). This inhibitor result further support the shRNA result that tumor cell migration is dependent on PAK1 in osteosarcoma cells.

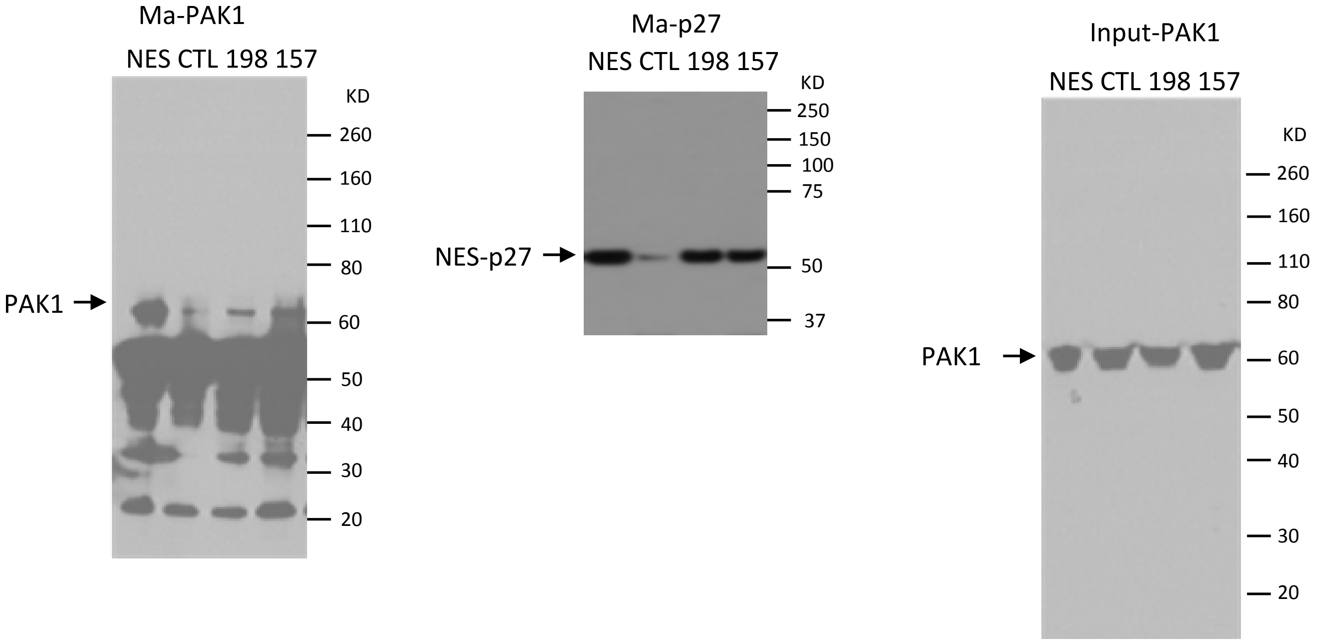

Ma-PAK1
NES CTL 198 157
KD
260
160
110
80
60
50
40
30
20
PAK1
Ma-p27
NES CTL 198 157
KD
250
150
100
75
50
37
NES-p27
Input-PAK1
NES CTL 198 157
KD
260
160
110
80
60
50
40
30
20
PAK1

Fig. 2B

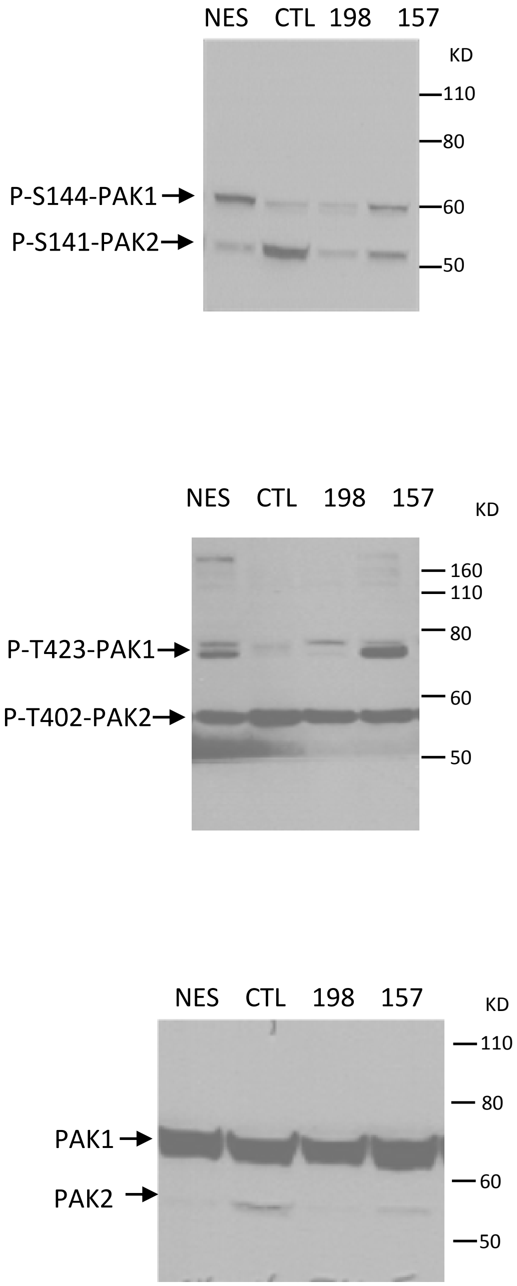
NES CTL 198 157
KD
110
80
P-S144-PAK1
60
P-S141-PAK2
50
NES CTL 198 157
KD
160
110
80
P-T423-PAK1
60
P-T402-PAK2
50
NES CTL 198 157
KD
110
80
PAK1
60
PAK2
50

Fig. 2D

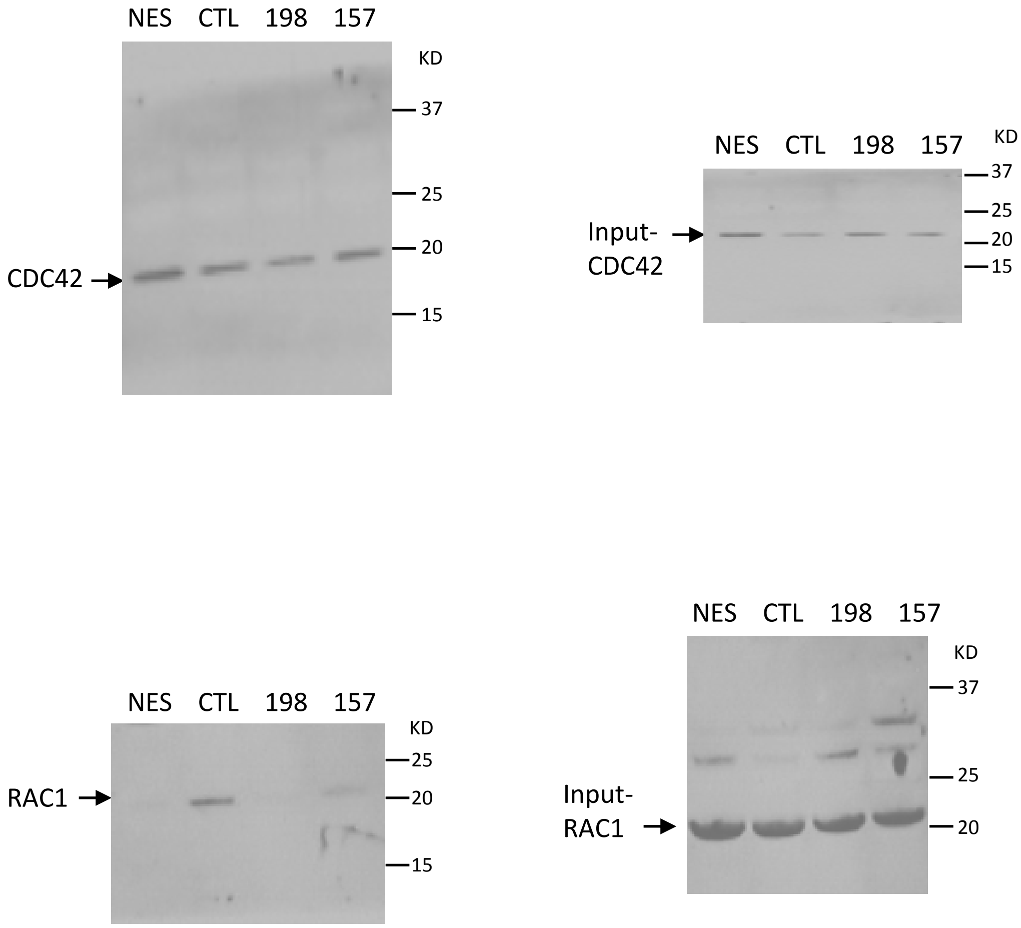
NES CTL 198 157
KD
37
25
20
15
CDC42
NES CTL 198 157
KD
37
25
20
15
Input-
CDC42
NES CTL 198 157
KD
25
20
15
RAC1
NES CTL 198 157
KD
37
25
20
Input-
RAC1

Fig. 2F

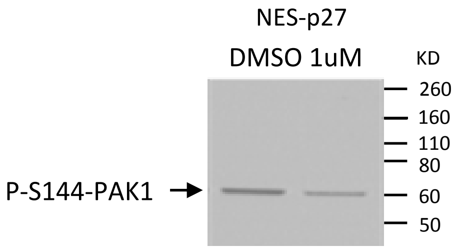
NES-p27
DMSO 1uM
KD
260
160
110
80
60
50
P-S144-PAK1

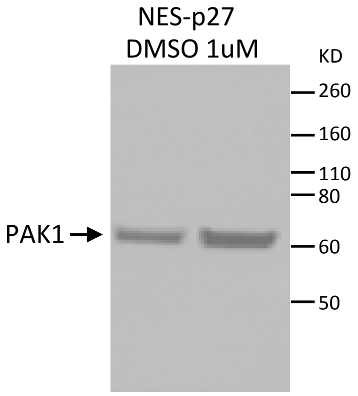
NES-p27
DMSO 1uM
KD
260
160
110
80
60
50
PAK1

Fig. 2H

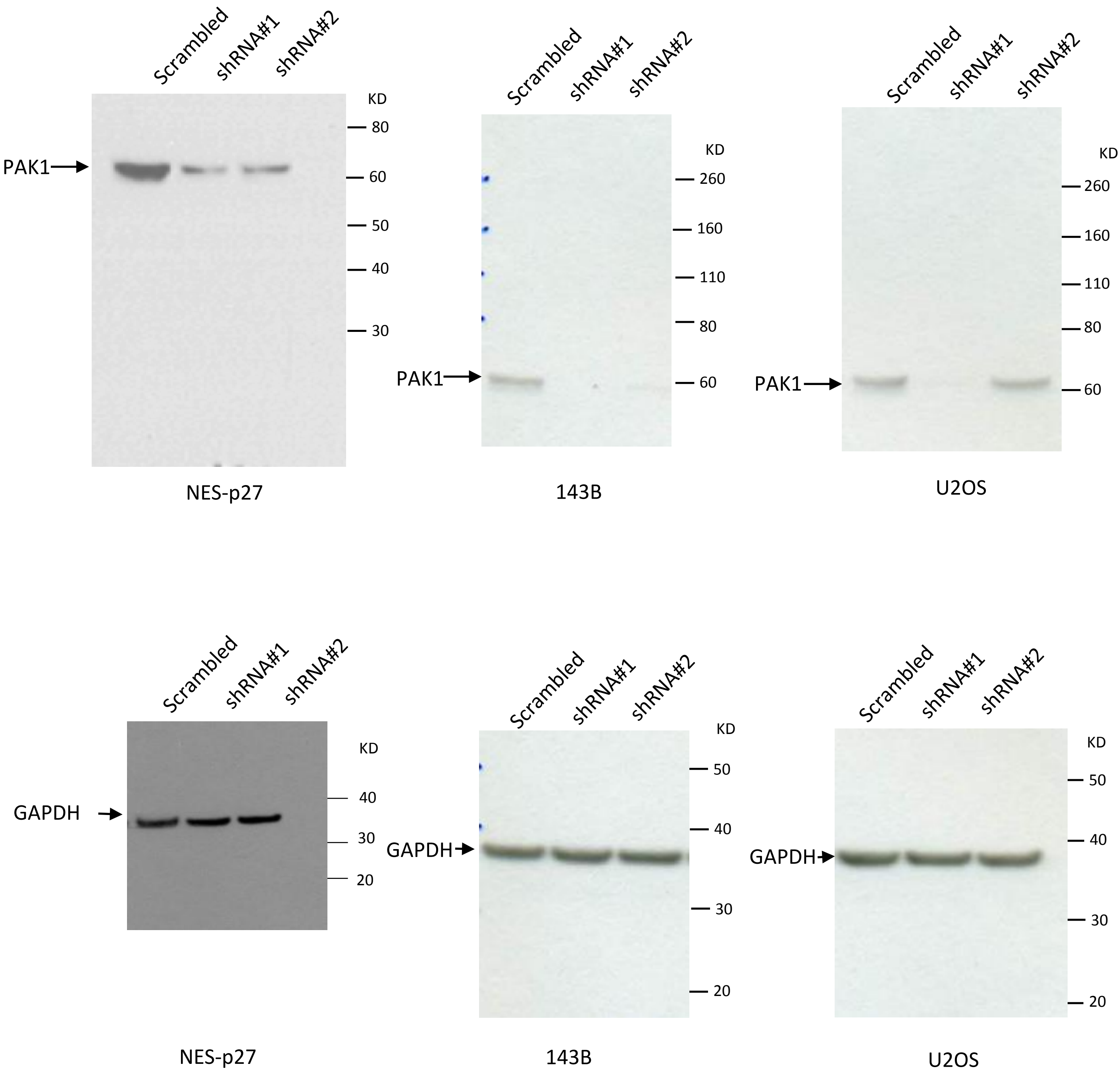
Scrambled
shRNA#1
shRNA#2
KD
80
60
50
40
30
PAK1
NES-p27
Scrambled
shRNA#1
shRNA#2
KD
260
160
110
80
60
PAK1
143B
Scrambled
shRNA#1
shRNA#2
KD
260
160
110
80
60
PAK1
U2OS
Scrambled
shRNA#1
shRNA#2
KD
40
30
20
GAPDH
NES-p27
Scrambled
shRNA#1
shRNA#2
KD
50
40
30
20
GAPDH
143B
Scrambled
shRNA#1
shRNA#2
KD
50
40
30
20
GAPDH
U2OS

Fig. 3A

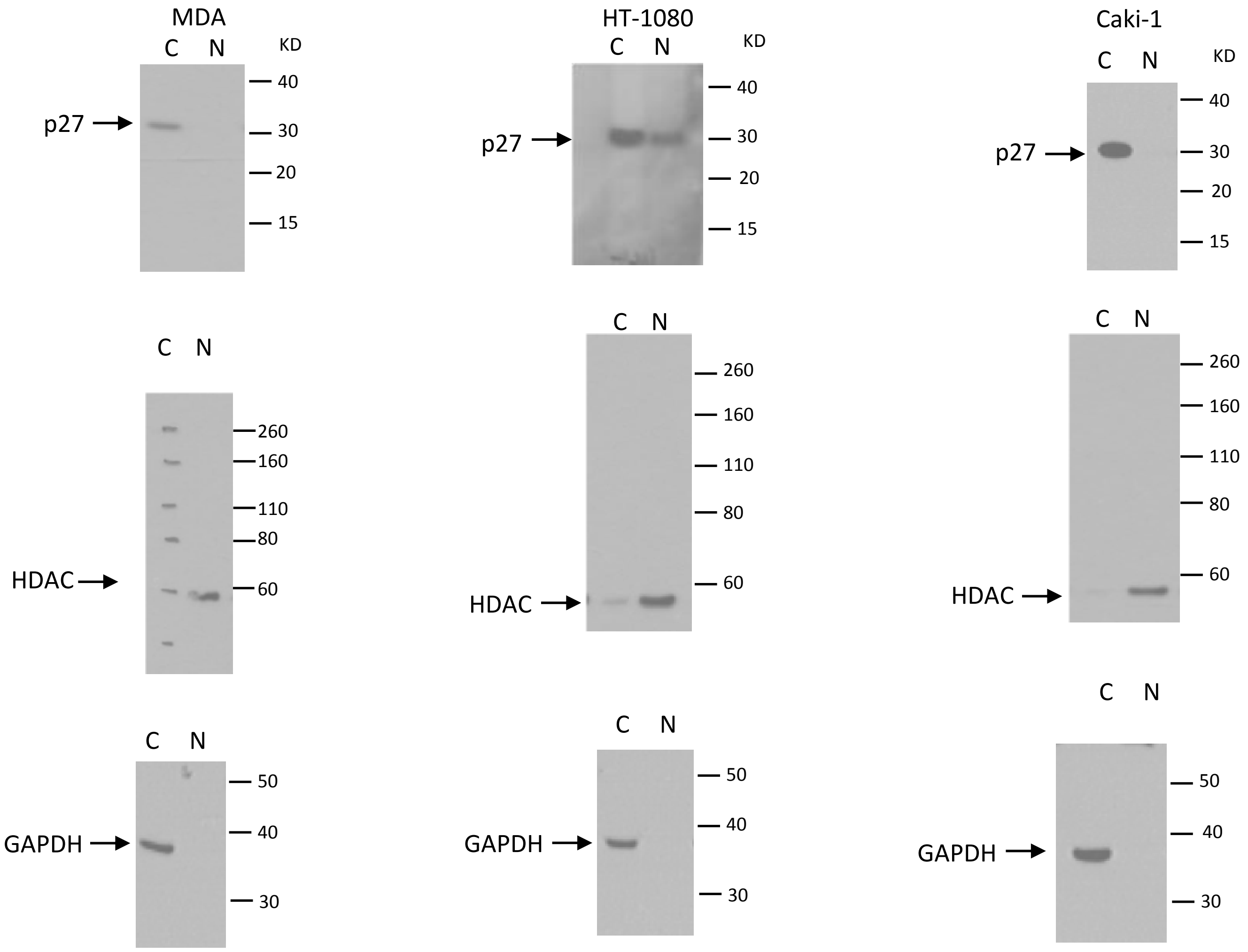
MDA
C N
KD
40
30
20
15
p27
HT-1080
C N
KD
40
30
20
15
p27
Caki-1
C N
KD
40
30
20
15
p27
C N
260
160
110
80
60
HDAC
C N
260
160
110
80
60
HDAC
C N
260
160
110
80
60
HDAC
C N
50
40
30
GAPDH
C N
50
40
30
GAPDH
C N
50
40
30
GAPDH

Fig. 6A

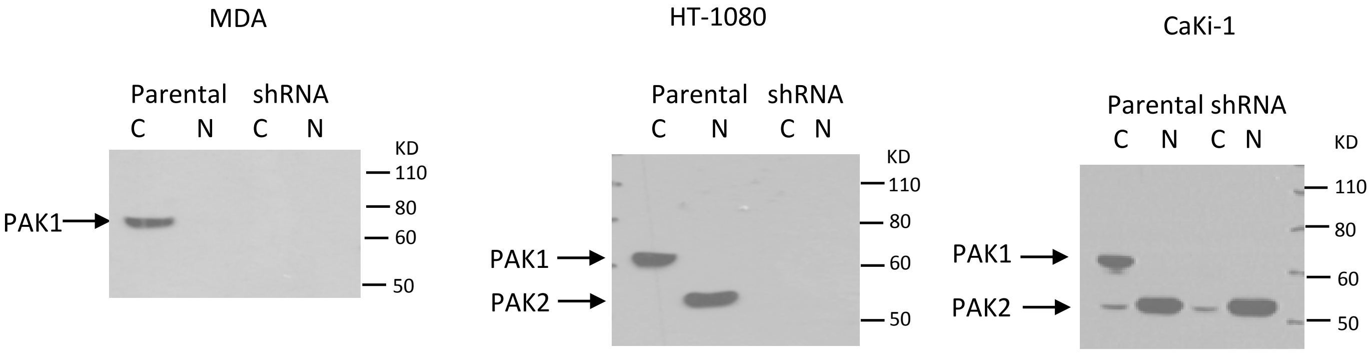
MDA
Parental shRNA
C N C N
KD
110
80
60
50
PAK1
HT-1080
Parental shRNA
C N C N
KD
110
80
60
50
PAK1
PAK2
CaKi-1
Parental shRNA
C N C N
KD
110
80
60
50
PAK1
PAK2

Fig. 6B

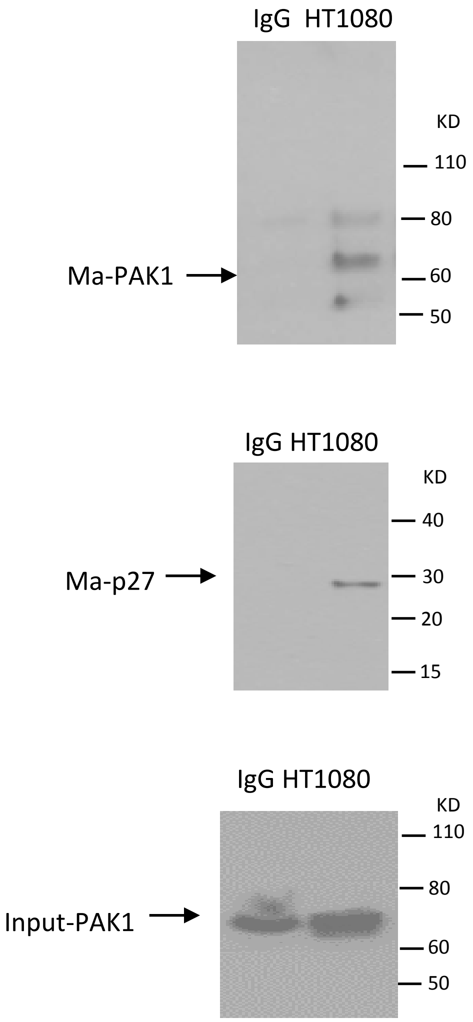
IgG HT1080
KD
110
80
Ma-PAK1
60
50
IgG HT1080
KD
40
Ma-p27
30
20
15
IgG HT1080
KD
110
80
Input-PAK1
60
50

Fig. S3A

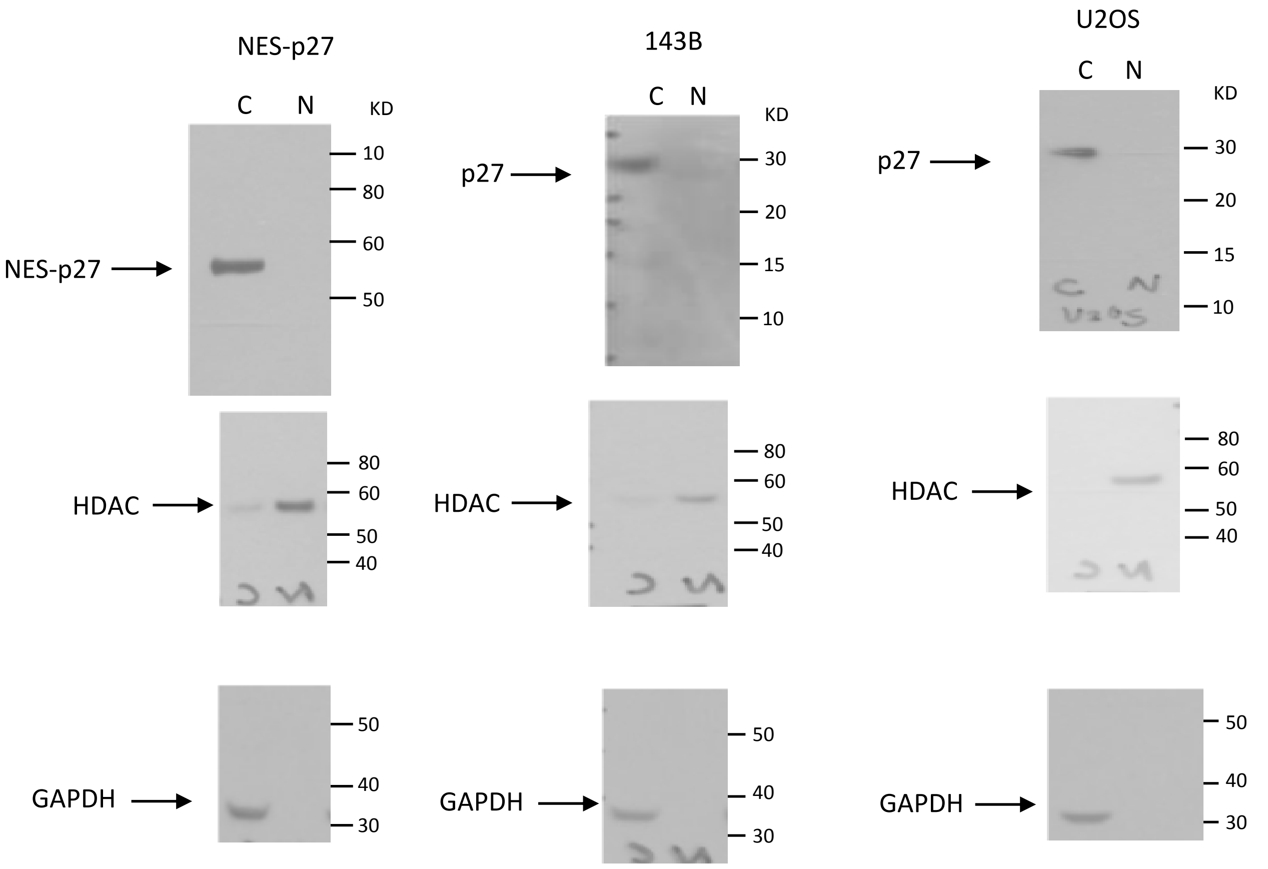
NES-p27
143B
U2OS
C
N
KD
NES-p27
p27
HDAC
GAPDH
10
80
60
50
30
20
15
40

Fig. S3C

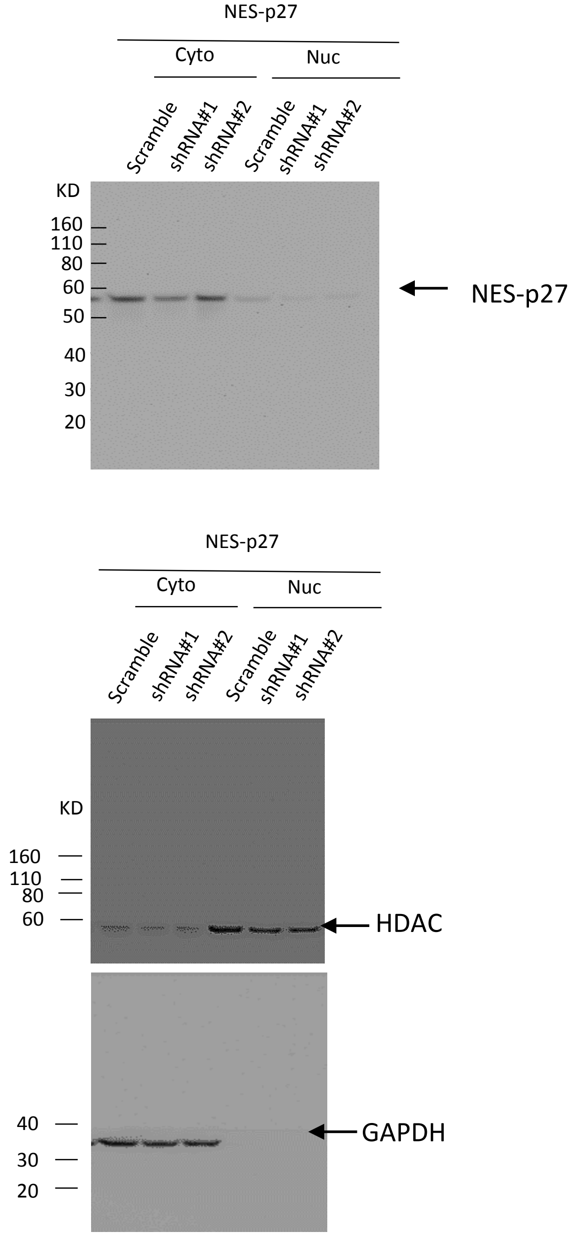
NES-p27
Cyto
Nuc
Scramble
shRNA#1
shRNA#2
Scramble
shRNA#1
shRNA#2
KD
160
110
80
60
50
40
30
20
NES-p27
NES-p27
Cyto
Nuc
Scramble
shRNA#1
shRNA#2
Scramble
shRNA#1
shRNA#2
KD
160
110
80
60
HDAC
40
30
20
GAPDH

Fig. S4

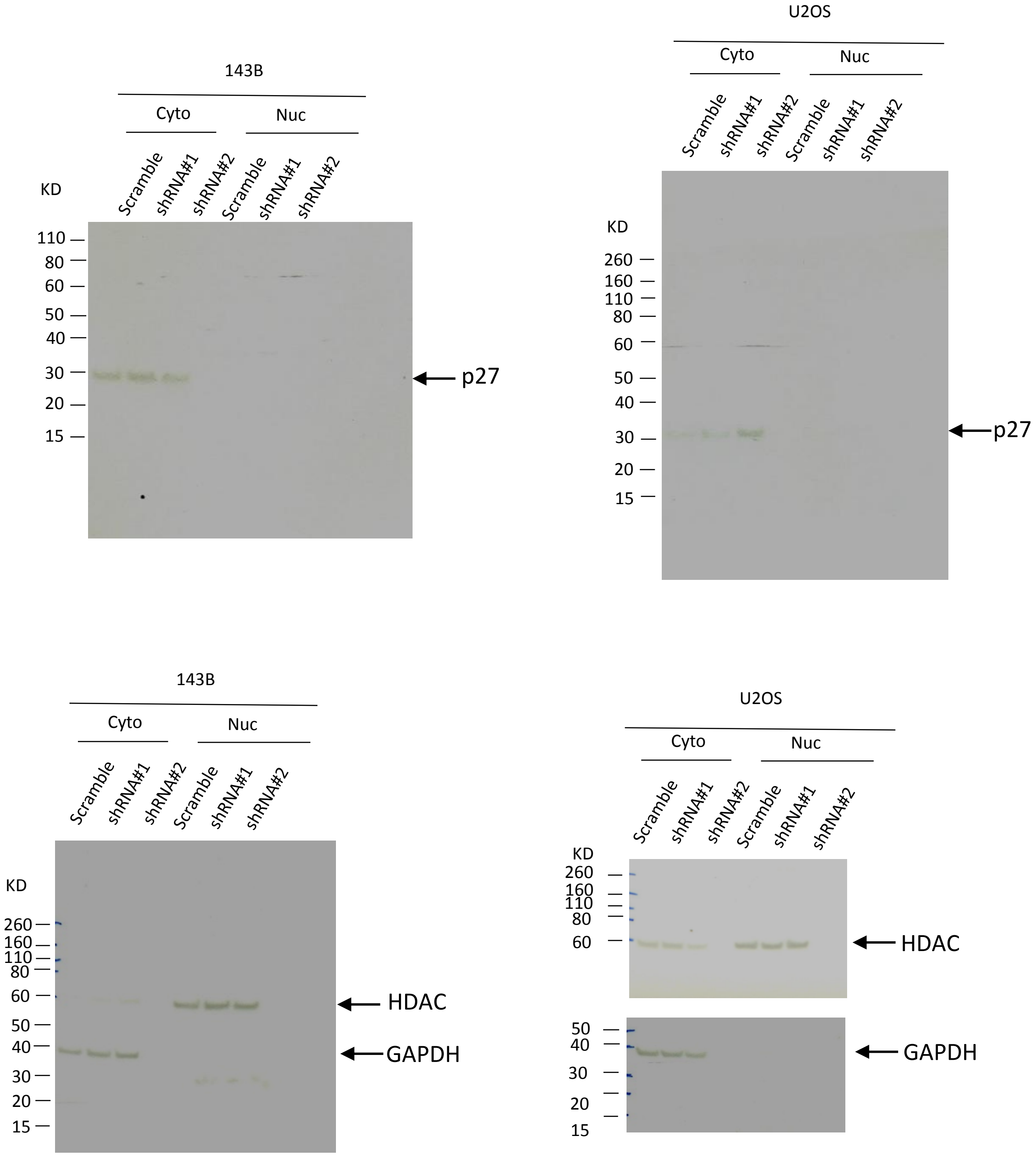
143B
Cyto
Nuc
Scramble
shRNA#1
shRNA#2
Scramble
shRNA#1
shRNA#2
KD
110
80
60
50
40
30
20
15
p27
U2OS
Cyto
Nuc
Scramble
shRNA#1
shRNA#2
Scramble
shRNA#1
shRNA#2
KD
260
160
110
80
60
50
40
30
20
15
p27
143B
Cyto
Nuc
Scramble
shRNA#1
shRNA#2
Scramble
shRNA#1
shRNA#2
KD
260
160
110
80
60
50
40
30
20
15
HDAC
GAPDH
U2OS
Cyto
Nuc
Scramble
shRNA#1
shRNA#2
Scramble
shRNA#1
shRNA#2
KD
260
160
110
80
60
HDAC
50
40
30
20
15
GAPDH

Fig. S4

Review Figure 1. Original Western blotting results for review. The uncropped original image of Western blotting of the Main Figures 2B, 2D, 2F (GTPase activity), 2H, 3A, 6A, 6B, and the Supplementary Figures S3A, S3C and S4 are provided for review.
